# ALK-Negative Anaplastic Large Cell Lymphoma: Current Concepts and Molecular Pathogenesis of a Heterogeneous Group of Large T-Cell Lymphomas

**DOI:** 10.3390/cancers13184667

**Published:** 2021-09-17

**Authors:** Sergio Pina-Oviedo, Carlos Ortiz-Hidalgo, Adrian Alejandro Carballo-Zarate, Alejandra Zarate-Osorno

**Affiliations:** 1 Department of Pathology, University of Arkansas for Medical Sciences, Little Rock, AR 72205, USA; 2 Department of Pathology, Hospital Médica Sur, Mexico City 14050, Mexico; cortizh@medicasur.org.mx; 3 Department of Pathology, Hospital Español, Mexico City 11520, Mexico; adrian_alejandroc@hotmail.com (A.A.C.-Z.); alejandra.zarateosorno@gmail.com (A.Z.-O.)

**Keywords:** T-cell lymphoma, anaplastic, CD30, JAK/STAT, differential diagnosis, cutaneous lymphoproliferative disorder, DUSP22, pathogenesis, breast implant

## Abstract

**Simple Summary:**

ALK- anaplastic large cell lymphoma (ALK- ALCL) is a rare subtype of CD30+ large T-cell lymphoma that typically affects older adults and has a poor prognosis. Recognition of its histopathologic spectrum, subtypes, and of other tumors that can resemble ALK- ALCL is crucial to avoid making a wrong diagnosis that could result in inappropriate treatment for a patient. In recent years, several important studies have identified recurrent molecular alterations that have shed light on the pathogenesis of this lymphoma. However, on the other hand, putting all this vast information together into a concise form has become challenging. In this review, we present not only a more detailed view of the histopathologic findings of ALK- ALCL but also, we attempt to provide a more simplified perspective of the relevant genetic and molecular alterations of this type of lymphoma, that in our opinion, is not available to date.

**Abstract:**

Anaplastic large cell lymphoma (ALCL) is a subtype of CD30+ large T-cell lymphoma (TCL) that comprises ~2% of all adult non-Hodgkin lymphomas. Based on the presence/absence of the rearrangement and expression of anaplastic lymphoma kinase (ALK), ALCL is divided into ALK+ and ALK-, and both differ clinically and prognostically. This review focuses on the historical points, clinical features, histopathology, differential diagnosis, and relevant cytogenetic and molecular alterations of ALK- ALCL and its subtypes: systemic, primary cutaneous (pc-ALCL), and breast implant-associated (BIA-ALCL)**.** Recent studies have identified recurrent genetic alterations in this TCL. In systemic ALK- ALCL, rearrangements in *DUSP22* and *TP63* are detected in 30% and 8% of cases, respectively, while the remaining cases are negative for these rearrangements. A similar distribution of these rearrangements is seen in pc-ALCL, whereas none have been detected in BIA-ALCL. Additionally, systemic ALK- ALCL—apart from *DUSP22*-rearranged cases—harbors *JAK1* and/or *STAT3* mutations that result in the activation of the JAK/STAT signaling pathway. The *JAK1/3* and *STAT3* mutations have also been identified in BIA-ALCL but not in pc-ALCL. Although the pathogenesis of these alterations is not fully understood, most of them have prognostic value and open the door to the use of potential targeted therapies for this subtype of TCL.

## 1. Introduction

Anaplastic large cell lymphoma (ALCL) is a subtype of T-cell lymphoma (TCL) composed of large cells and a characteristic strong and diffuse expression of the activation marker CD30. As its name points out, this lymphoma is composed of large epithelioid cells with anaplastic features, namely significant atypia and marked pleomorphism. Curiously, the scientific community recognizes the descriptive term “ALCL” as a TCL despite the fact that there is no reference to its T-cell lineage in the name, as is the case for anaplastic large “B-cell” lymphoma.

ALCL is divided into two major groups based on the presence/absence of the rearrangement and of the expression of the anaplastic lymphoma kinase (ALK) into ALK-positive (ALK+) and ALK-negative (ALK-), with each group roughly comprising 50% of cases [1]. ALK+ ALCL is more common in the pediatric population and has a good overall prognosis (overall survival 70–80%), whereas ALK- ALCL is more common in adults and has a more aggressive behavior (overall survival 40–60%), although prognosis may also be related to the age of presentation rather than to ALK expression [2,3]. In contrast to ALK+ ALCL where the expression of ALK and CD30 virtually excludes any other diagnostic possibility regardless of the morphology, the distinction between ALK- ALCL and other large TCLs with CD30 expression is difficult.

Based on its location and extension of involvement, ALK- ALCL is currently classified into (1) systemic, (2) primary cutaneous, and (3) breast implant-associated ALCL [1]. Recent molecular studies have identified diverse genetic alterations in ALK- ALCL that convey different prognoses and have different underlying pathogenetic mechanisms, obviating the heterogeneity of this group of lymphomas. It is very likely that in the coming years systemic ALK- ALCL may be further divided into molecular subtypes according to the presence/absence of genetic abnormalities. In this review, we discuss historical points, clinical features, histopathology, differential diagnoses, and relevant genetic and molecular alterations of all subtypes of ALK- ALCL. Treatment modalities are out of the scope of this review and are not discussed here.

## 2. Systemic ALK- ALCL

### 2.1. Definition

This lymphoma is defined as the involvement of the lymph nodes, bone marrow, and/or extranodal organs by ALK- ALCL at initial presentation. Primary cutaneous ALCL (pc-ALCL) and breast implant-associated ALCL (BIA-ALCL) are excluded from this group (discussed below), but it is difficult—if not impossible—to distinguish systemic disease from multiorgan spread from these two subtypes and only a prior clinical history of cutaneous or breast-implant associated disease may point to this possibility.

### 2.2. Historical Aspects

Most cases of ALCL were categorized in the past as non-hematopoietic neoplasms or as tumors of histiocytic or reticulum cell origin (histiocytic lymphoma, malignant histiocytosis, etc.) [4]. It was not until the application of immunohistochemistry and the capability to grow cultures of Reed-Sternberg cells from classic Hodgkin lymphoma (CHL) by Volker Diehl, Harald Stein, and colleagues in Germany that the discovery of the CD30 antigen was possible in 1982 [5]. This antigen was abundant in Reed-Sternberg cells and received the name “Ki-1” (“Ki” from “Kiel” and “1” alluding to the number of the antibody clone, i.e., the other well-known antigen discovered in these studies was Ki-67). In 1985, H. Stein et al. recognized that a group of anaplastic hematopoietic tumors with particular nodal involvement were positive for Ki-1 (later designated CD30), HLA-DR, and frequently positive for ≥1 T-cell markers (few cases had a B-cell phenotype) and designated these neoplasms “ALCL” or “anaplastic Ki-1 large cell lymphoma” [6]. ALCL was then the preferred name for this neoplasm in the Kiel classification of 1988 as well as in the Revised European American Lymphoma (REAL) classification of 1994 [7,8]. In the REAL classification, ALCL was recognized as a tumor of the T-cell or a “null” phenotype different from CD30+ large B-cell lymphomas.

Since there could not be an ALK- ALCL without ALK, one could say that the discovery of this lymphoma technically did not occur until 1994 when Stephan Morris, Thomas Look and colleagues at that time at Saint Jude Children’s Hospital in Memphis, TN, U.S., discovered the genes involved in the translocation t(2;5)(p23;q35) and recognized a novel tyrosine kinase in chromosome 2 that was called anaplastic lymphoma kinase (ALK) [9]. ALK- ALCL was included as a provisional entity in the 2008 World Health Organization (WHO) classification and is currently classified as a distinct entity in the current 2017 WHO revised 4th edition [1,10]. In the last few decades, most of the breakthrough genetic studies in relation to ALK- ALCL have come from several of the authors of past and current WHO classifications (Falini B., Pileri S., Delsol G., Stein H., Jaffe, E., among several others) and interinstitutional TCL study groups. During the last decade Andrew Feldman and his group at the Mayo Clinic in Rochester, MN, U.S. have discovered novel and seminal findings that have opened the door to new ways of classifying and possibly treating these groups of heterogeneous TCLs.

### 2.3. Epidemiology, Risk Factors, and Clinical Features

TCLs comprise between 10 to 15% of all non-Hodgkin lymphomas [11,12]. ALK+ and ALK- ALCL combined represent ~2% of all adult non-Hodgkin lymphomas and are the fourth most common TCL (~10%) after peripheral TCL, not otherwise specified (PTCL, NOS), mycoses fungoides, and angioimmunoblastic TCL [11,12].

Most individuals affected by ALK- ALCL are adults between 40 to 65 years of age and men are slightly more commonly affected than women (M:F ratio 1.5:1) [13]. They usually present at advanced stages of the disease with systemic B-symptoms, multiple lymphadenopathies, and organomegaly. ALK- ALCL involves the lymph nodes in ~50% of cases whereas extranodal involvement is less frequent [14,15]. Relatively common extranodal sites of involvement include the skin (excluding pc-ALCL), soft tissue, the liver and the lungs, whereas rare sites of involvement include the oropharynx, the gastrointestinal tract, the orbit, the brain, and the testes [3,16,17,18,19,20,21,22,23]. Skin lesions may be solitary or multiple in the form of papules, nodules, or tumors. The involvement of other organs usually occurs in the form of a mass. Leukemic presentation is very rare in ALK- ALCL and it portends a bad prognosis [24,25,26,27].

Based on data from the InterLymph Non-Hodgkin Lymphoma Subtypes Project, textile workers and electrical fitters have a significantly increased risk of ALCL (OR of 4.08 and 2.60, respectively) but according to the authors the sample size was modest and further information on ALK status was not sufficient for more definitive conclusions to be made [28]. Similarly, there are not enough data to establish an association between autoimmune disorders or an immunocompromised status and the development of ALK- ALCL [13]. Importantly, BIA-ALCL develops in the setting of long-standing textured breast implants (discussed below) [29,30,31].

In contrast to other TCLs, ALK- ALCL does not have a frequent association with infection by the Epstein-Barr virus (EBV) and only exceptional EBV+ cases have been reported in the literature [32,33]. No association with infection by the human T-cell leukemia virus type 1 (HTLV-1) or the human immunodeficiency virus (HIV) has been recognized hitherto.

### 2.4. Pathology

**Figure 1:**
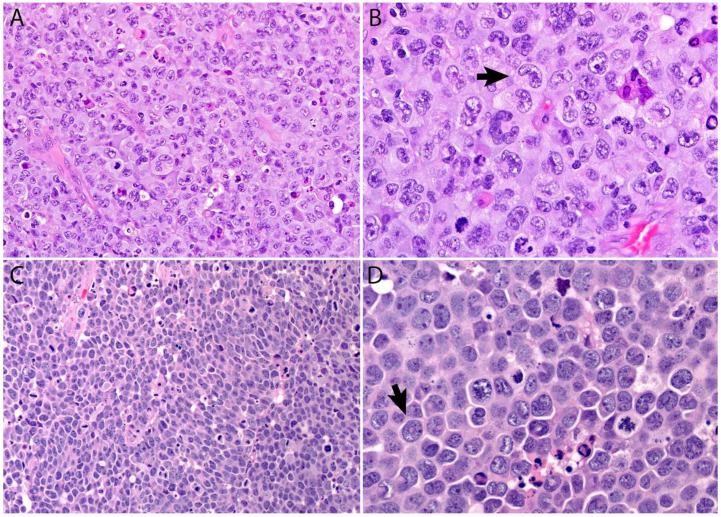
(**A**,**B**) ALCL is a neoplasm composed of sheets of large pleomorphic/anaplastic tumor cells with abundant cytoplasm. Occasional multinucleated cells and Reed-Sternberg-like cells are seen. (**B**) “Hallmark” cells are characteristic of this neoplasm (arrow). (**C**,**D**) Most cases of ALK- ALCL with the *DUSP22* rearrangement have a more monotonous appearance and are less pleomorphic. This case has a particular starry-sky appearance. (**D**) Cells with central nuclear pseudoinclusions or a “doughnut” nuclear shape are commonly seen (arrow).

A lymph node involved with ALCL is tan-white to pale-pink with variable hemorrhage and necrosis. These same features are seen in a mass involving an extranodal site. As defined by the WHO in 2017, ALK- ALCL is a “CD30+ T-cell neoplasm that is not reproducibly distinguishable on morphologic grounds from ALK+ ALCL, but lacks (ALK) protein expression” [1]. Therefore, the morphologic features described below are seen in both ALK+ and ALK- ALCL (common or classic variant). These tumors are composed of sheets of large epithelioid cells with variable degrees of pleomorphism and anaplasia (Figure 1A,B). The lymphoma cells have an eccentric nucleus with vesicular to moderately condensed chromatin, a prominent nucleolus, and an abundant eosinophilic or amphophilic cytoplasm with a prominent Golgi zone observed as a clear or deeply eosinophilic paranuclear region. The characteristic cells of ALCL have a horseshoe-shaped nucleus (first called “hallmark” cells by Benharroch and Delsol in 1998 [34]) and are present in all cases in variable amounts (Figure 1A,B). In addition, tumor cells may be binucleated (Reed-Sternberg-like), may have a ring-shaped nucleus with a central pseudoinclusion containing cytoplasm (“doughnut” cells), or may be multinucleated with nuclei arranged in a wreathlike configuration (Touton-like cells). Cells with an “embryoid” nucleus may also be seen. Even though morphology cannot reliably distinguish between ALK+ and ALK- ALCL, certain features might suggest that a case is ALK-, including the presence of plasmablastic features, and/or the presence of a starry-sky pattern (Figure 1C,D). Similarly, cases of ALK- ALCL harboring *DUSP22* rearrangements (see the molecular section) are more likely to contain “doughnut” cells, less pleomorphism, and a sheet-like growth with an insignificant number of background inflammatory cells (Figure 1C,D) [35]. Nevertheless, ALK immunohistochemistry is mandatory to confirm the diagnosis.

**Figure 2:**
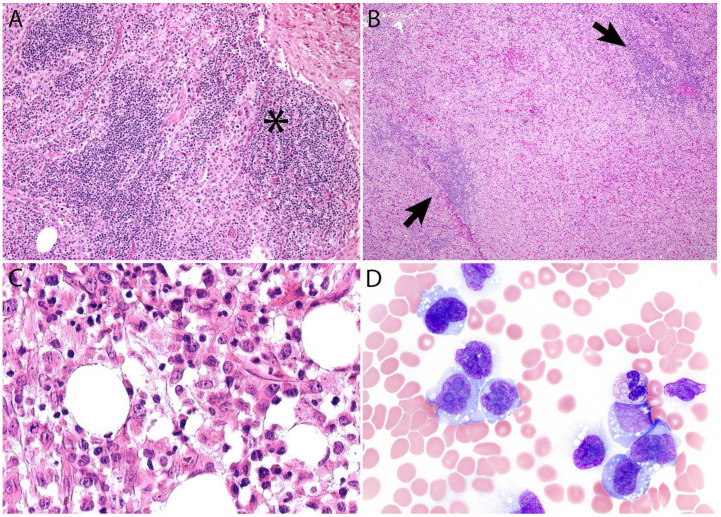
(**A**) Sinusoidal distribution of ALCL mimicking metastatic carcinoma (asterisk). (**B**) ALCL with marked expansion of the paracortical area and subtotal effacement of a lymph node. The arrows point to residual compressed lymphoid follicles. (**C**) ALK- ALCL involving bone marrow. (**D**) Leukemic ALK- ALCL mimicking monocytic leukemia. The neoplastic cells are intermediate to large with an irregular to reniform nucleus (“hallmark” cells) and moderate to abundant vacuolated cytoplasm.

Additional features include the presence of frequent mitoses and variable necrosis. Nodal involvement demonstrates a characteristic sinusoidal distribution of the tumor cells mimicking metastatic carcinoma, metastatic melanoma, or histiocytic sarcoma (Figure 2A). In cases with partial nodal involvement, the tumor cells are preferentially located in the interfollicular region (Figure 2B).

Bone marrow involvement may be nodular and readily identified on hematoxylin and eosin stain (Figure 2C); however, interstitial spread may be subtle, and the tumor cells may be missed if CD30 immunohistochemistry is not performed. The leukemic presentation of ALK- ALCL is very rare (more characteristic of ALK+ ALCL small cell variant) and when it is present, the tumor cells exhibit similar features to those described above and usually contain cytoplasmic vacuoles and/or few azurophilic granules (Figure 2D).

**Figure 3:**
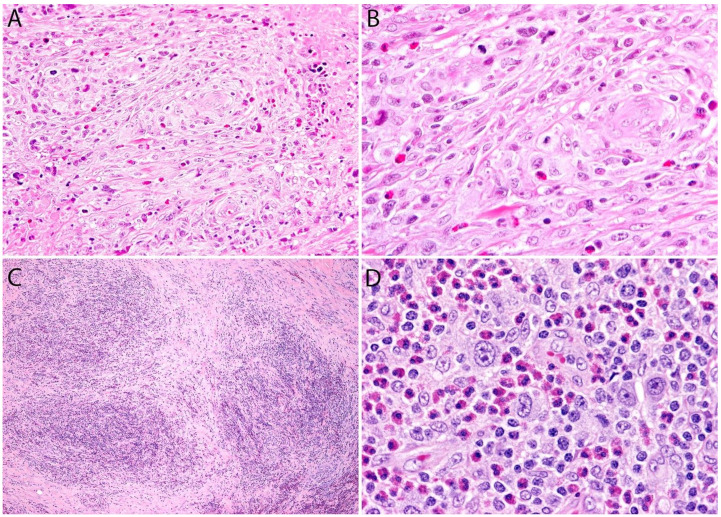
Variants of systemic ALK- ALCL. (**A**,**B**) Sarcomatoid ALCL. Note the perivascular arrangement and the focal areas of necrosis at the periphery. Occasional “hallmark” cells are seen. (**C**,**D**) “Hodgkin-like” ALCL. This variant is identical to nodular sclerosis classic Hodgkin lymphoma and can only be distinguished by immunohistochemistry. The tumor cells in both cases were positive for CD30, CD4, and other T-cell markers, and were negative for CD8, CD15, PAX5, and ALK (not shown in figure).

Although the morphologic variants of ALCL are not officially recognized in the ALK- group [1,36], it is still possible to make a diagnosis of ALK- ALCL in cases that have areas with a classic/common morphology adjacent to variant features, namely signet-ring cells, sarcomatoid morphology, or a background rich in neutrophils (“pyogenic-like”) or eosinophils [14,37]. However, this may not be the case for the small cell variant—and to some extent also the lymphohistiocytic variant—that can only be diagnosed as such using ALK immunohistochemistry [14,36,37]. Sarcomatoid ALCL consists of a tumor with a variable proportion of pleomorphic epithelioid cells and spindle cells arranged in short fascicles or in a storiform pattern and a myxoid background with a few scattered small lymphocytes, neutrophils, and/or eosinophils [37,38] (Figure 3A,B). The tumor cells tend to arrange around blood vessels with partial infiltration into the vascular wall. A rare form of ALCL morphologically identical to nodular sclerosis classic Hodgkin lymphoma (CHL) is referred to as ALCL with a “Hodgkin-like pattern” [37,39] (Figure 3C,D).

#### Immunohistochemistry

**Figure 4:**
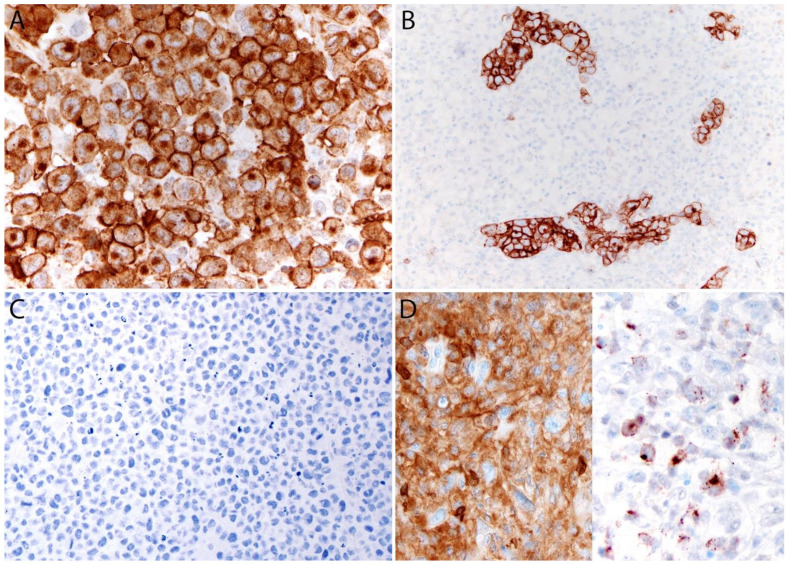
Immunohistochemistry in systemic ALK- ALCL. (**A**) CD30 (120 kDa glycoprotein) is strongly and diffusely positive in the tumor cells with a membranous and Golgi dot-like pattern. The Golgi labelling corresponds to the location of the CD30 protein precursor of 90 kDa. (**B**) CD30 highlights the sinusoidal distribution in a partially involved lymph node. (**C**) The ALK immunostain is negative. (**D**) Characteristic co-expression of CD4 (left) and cytotoxic markers, such as granzyme B (right).

**Figure 5:**
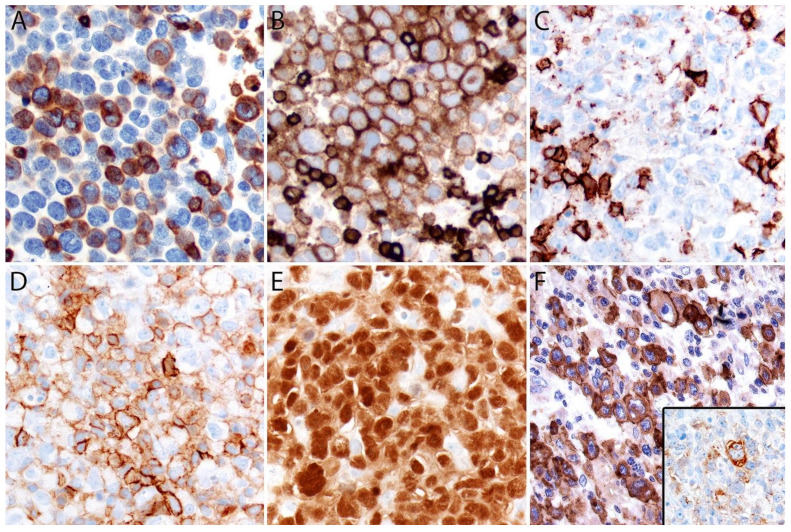
Immunohistochemistry in systemic ALK- ALCL. (**A**) Variable expression of CD3. (**B**) Positive CD5. (**C**) CD8 is negative in the large lymphoma cells and positive in background cytotoxic T-cells. (**D**) Variable CD45. (**E**) Strong MUM1 expression. (**F**) EMA is positive in ~40% of cases. Rare cases can express ≥1 cytokeratin (**F**, inset) that along with a positive EMA could be misinterpreted as carcinoma if ALCL is not considered in the differential diagnosis.

By definition, ALK- ALCL is strongly and diffusely positive for CD30 (>75% of cells) and negative for ALK (Figure 4A–C). CD30 decorates the lymphoma cells with a membranous and paranuclear dot (Golgi) pattern (Figure 4A,B). For uncertain reasons, this lymphoma shows a peculiar paradoxical expression of CD4 and of cytotoxic markers, namely T-cell intracellular antigen-1 (TIA-1), granzyme B, and perforin (Figure 4D). However, it is now well-recognized that *DUSP22-*rearranged cases are usually negative for cytotoxic molecules [40] (see section on molecular alterations). The T-cell antigens CD2, CD3, CD5, CD7, and CD8 are variably expressed (Figure 5A–C) and cases that lack all T-cell antigens are referred to as having a “null” phenotype [36,41,42]. CD56 and CD45/leukocyte common antigen are variably expressed (Figure 5D), and some cases may be negative for CD45, whereas CD43 is expressed in most cases [3,36,42]. Clusterin and multiple myeloma 1/interferon regulatory factor 4 (MUM1/IRF4) are usually positive (Figure 5E), and in contrast to ALK+ ALCL, only ~40% of cases are positive for the epithelial membrane antigen (EMA) [3] (Figure 5F). ALK- ALCL is negative for CD15, B-cell markers (CD19, CD20, CD79a, PAX5), and for EBER by in situ hybridization. However, pathologists should be aware that rare cases may be positive for CD15 and/or PAX5, the latter due to extra copies of the *PAX5* gene locus [43]. Likewise, *bona fide* cases of EBER+ ALK- ALCL have been reported but they are very rare [32,33]. Unusual cytokeratin expression has also been reported in rare cases [44,45,46] (Figure 5F inset). ALK- ALCL cases with the *TP63* rearrangement are positive for p63 by immunohistochemistry (see section on molecular alterations) [40,47]. Loss of CD30 has been described in sporadic cases after the use of brentuximab-vedotin (anti-CD30) therapy [48,49,50,51].

### 2.5. Differential Diagnosis

#### 2.5.1. Hematopoietic Tumors

The differential diagnosis of ALK- ALCL includes ALK+ ALCL, other large PTCLs, CHL, anaplastic large B-cell lymphoma, monocytic sarcoma, and histiocytic sarcoma (See also Table 1). The distinction between pc-ALCL, BIA-ALCL, and systemic ALK- ALCL requires a well-documented history and clinical correlation. These differences are addressed in the respective sections.

##### ALK- vs. ALK+ ALCL

**Figure 6:**
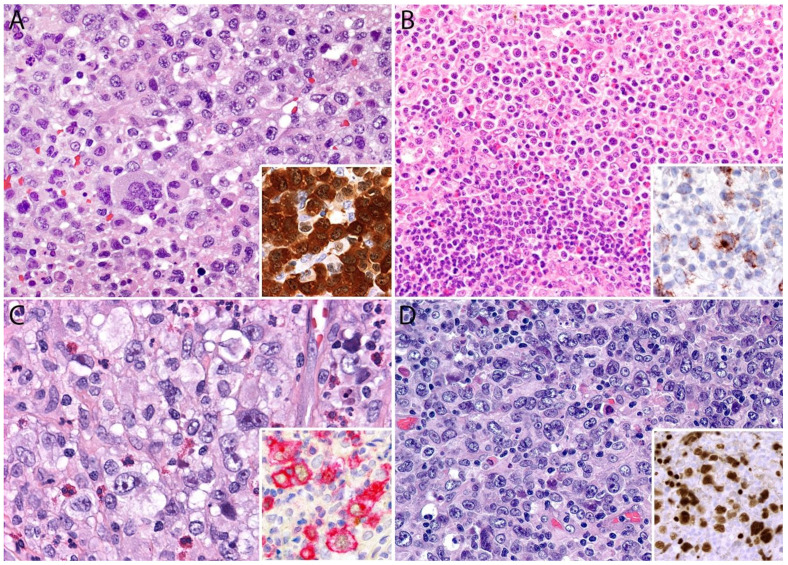
Differential diagnosis of systemic ALK- ALCL. (**A**) The morphology of ALK+ ALCL is indistinguishable from that of ALK- ALCL; however, the ALK immunostain is positive (inset). (**B**) Peripheral T-cell lymphoma, not otherwise specified, with large cells. The tumor cells have a paracortical distribution but are monotonous and not as pleomorphic as seen in ALCL. In addition, CD30 is only focally positive (inset). (**C**) Classic Hodgkin lymphoma. Reed-Sternberg cells are positive for CD30 (red) and dim PAX5 (inset, CD30/PAX5 double immunolabeling). (**D**) Anaplastic large B-cell lymphoma. The tumor cells are strongly positive for PAX5 (inset).

As mentioned previously, ALK+ and ALK- ALCL are morphologically indistinguishable and both conditions are positive for CD30. In contrast to its ALK+ counterpart, ALK- ALCL is more frequently positive for CD3 and clusterin and is more likely to be positive for CD15 and PAX5. Cases with the *DUSP22* rearrangement are usually negative for cytotoxic markers and EMA and demonstrate LEF1 overexpression [40,52]. However, the presence or absence of these markers only suggests—and does not confirm—the diagnosis. An ALK immunostain is the only method to distinguish these entities (Figure 6A). A detailed explanation of the different patterns of subcellular localization of ALK in ALK+ ALCL is out of the scope of this review. Fluorescence in situ hybridization (FISH) studies for *ALK* rearrangement are not needed if ALK immunohistochemistry is negative [1,14].

##### ALK- ALCL vs. PTCL, NOS

The distinction between ALK- ALCL and PTCL, NOS with large cells and CD30 expression (seen in ~10% of PTCL, NOS) is not only challenging but it is also prone to subjectivity. In general, a diagnosis of CD30+ PTCL, NOS with large cells should be made when the morphology and the immunoprofile of the tumor do not meet the features previously described for ALK- ALCL, namely, (1) a lack of sheets of large anaplastic cells, (2) a lack of “hallmark” cells, (3) a lack of a sinusoidal pattern, and (4) CD30 expression in <75% of lymphoma cells (Figure 6B). This also applies to lymphomas with a classic/common ALCL morphology that are negative, weak, or only focally positive for CD30 (<75% of tumor cells). The expression of CD8 and/or a lack of cytotoxic markers does not preclude a diagnosis of ALK- ALCL if the rest of the diagnostic features are present. Rearrangements of *DUSP22* or *TP63* genes favor a diagnosis of ALK- ALCL over CD30+ PTCL, NOS, but they are not confirmatory as a small subset of PTCL, NOS may also harbor these genetic alterations [53]. Predictive models and gene classifiers appear to be able to discern ALK- ALCL from PTCL, NOS but these studies are not widely available for routine practice and are costly (see section of molecular alterations).

##### ALK- ALCL with a “Hodgkin-like Pattern” vs. CHL

In CHL, the Reed-Sternberg cells are usually scattered throughout a polymorphic background and this feature is also seen in ALK- ALCL with a “Hodgkin-like pattern” [39]. On the other hand, sheets of Reed-Sternberg cells mimicking ALCL are seen in the syncytial variant of nodular sclerosis CHL. Some morphologic clues that may favor ALCL over CHL are the presence of tumor cells in sinusoids and a decreased paucicellular inflammatory background, but these features may occasionally be seen in CHL. Therefore, a reliable morphologic distinction of both entities is challenging—if not impossible—without the use of immunohistochemistry (Figure 6C). Reed-Sternberg cells are positive for CD30, CD15 (60–70% of cases), PAX5 (weak) (Figure 6C), and MUM1, and are negative for CD45, EMA, CD20, and other B-cell markers. EBER is variably positive (~20% in nodular sclerosis CHL). The diagnosis of CHL is straightforward when this immunophenotype is present. However, it is recognized that ~5% of CHLs may show a T-cell phenotype with the expression of ≥1 T-cell antigen(s) and cytotoxic markers [54]. To further complicate the issue, ALK- ALCL may show expression of CD15 and PAX5 overlapping with the immunophenotype of CHL [43,55]. In this scenario, the utilization of several other antibodies, such as additional T-cell markers, clusterin, EMA, and EBER, is required to best classify a case. However, this still may not be sufficient to arrive at a final diagnosis and an analysis for clonal *IGH* and *TCR* gene rearrangements may prove useful in these cases. More than 90% of ALCLs show a clonal *TCR* rearrangement irrespective of the T-cell antigen status [41]. Nevertheless, the interpretation of the *TCR* gene rearrangement should also be performed with caution since 1–2% of CHLs may demonstrate *TCR* clonality [56,57]. One study has shown that an immunohistochemical classifier using MDC/CCL22, CD83, STAT3, and TUBB2B has an accuracy of 97% to distinguish between CHL (all four markers positive) and ALK- ALCL (all four markers negative) [58], but these antibodies are not available in routine pathology practice. Another study has shown that 57% (13/28) of ALK- ALCL cases express pSTAT3 by immunohistochemistry and this may also be an additional useful marker to distinguish between CHL and ALK- ALCL [59]. In the experience of experts in the field, CHL with a T-cell phenotype and/or positive *TCR* clonality is best classified as a PTCL, NOS rather than ALK- ALCL [1,36,42]. We wonder if such unusual cases might be considered part of a spectrum as once suggested by others [60] or if they might represent a “gray zone lymphoma with intermediate features between ALCL and T-cell CHL”. If intermediate CHL and B-cell lymphomas do occur, it is not surprising that intermediate CHL and TCLs may also exist. An important clinical consideration is that ALCL does not follow a contiguous spread to lymph nodes in the characteristic way that CHL does.

##### ALK- ALCL vs. Large B-Cell Lymphoma, Monocytic Sarcoma and Histiocytic Neoplasms

**Figure 7:**
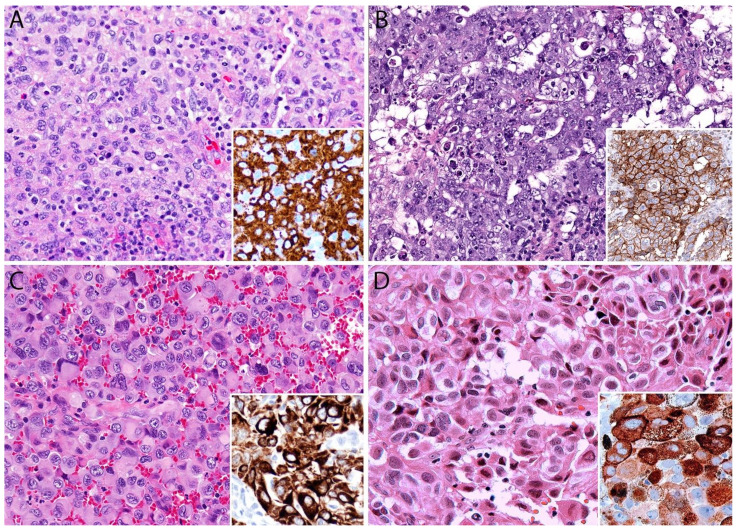
Differential diagnosis of systemic ALK- ALCL. (**A**) Histiocytic sarcoma resembling ALCL. The tumor cells are positive for CD163 (inset), and negative for CD30 or T-cell markers (not shown in Figure). (**B**) Embryonal carcinoma with solid and pseudoglandular patterns. Note the deeply basophilic cytoplasm, anaplastic features, and abundant apoptosis. The tumor is strongly positive for CD30 (inset), cytokeratin, and OCT3/4 (not shown in Figure). (**C**) Epithelioid sarcoma mimicking ALCL. The tumor is positive for cytokeratin (inset) and ERG, while negative for INI1 (not shown in Figure). (**D**) Epithelioid melanoma metastatic to a lymph node. The neoplastic cells are positive for Melan-A (inset).

The distinction between ALK- ALCL and anaplastic large B-cell lymphoma, histiocytic sarcoma, and monocytic sarcoma is readily established by immunohistochemistry. The expression of strong and diffuse PAX5 and CD20 along with other B-cell markers (CD19, CD79a) or B-cell related transcription factors (OCT2, BOB.1) supports anaplastic large B-cell lymphoma and excludes ALK- ALCL (Figure 6D). Likewise, the expression of monocytic or macrophage-related antigens, namely CD4, CD14, CD68, CD163, lysozyme, and a lack of CD30 and T-cell markers supports a monocyte/histiocyte origin and excludes ALK- ALCL (Figure 7A). Unlike ALK+ ALCL, only rare cases of ALK- ALCL express CD13 and/or CD33 [61] and a strong expression of CD30 and other T-cell antigens should be sufficient to establish a diagnosis. Langerhans cell histiocytosis and Rosai-Dorfman disease do not pose a problem of interpretation with ALCL since they do not feature significant atypia and have a characteristic morphology.

#### 2.5.2. Non-Hematopoietic Tumors

The differential diagnosis includes carcinoma, embryonal carcinoma, melanoma, and other tumors with epithelioid and rhabdoid features, namely alveolar soft part sarcoma, epithelioid sarcoma, rhabdoid tumors, and the rare epithelioid variant of myofibroblastic inflammatory sarcoma. This differential diagnosis is applicable to primary extranodal malignancies as well as for lymph node metastases. The sarcomatoid variant of ALK- ALCL should be distinguished from sarcomatoid carcinoma, high grade spindle cell sarcomas, and inflammatory pseudotumor (See also Table 2). Fortunately, a proper panel of antibodies is sufficient to distinguish ALK- ALCL from all these tumors, as detailed below.

##### ALK- ALCL vs. Carcinoma and Embryonal Carcinoma

Although carcinomas are readily distinguished by morphology from ALCL given the lack of “hallmark” cells and glandular or squamous differentiation, poorly differentiated cases may show an overlapping morphology with this type of TCL. ALK- ALCL is negative for cytokeratins and the germ cell markers OCT3/4 and SALL4. Embryonal carcinoma usually demonstrates pseudoglandular, papillary, or alveolar patterns that do not suggest lymphoma. However, embryonal carcinoma with a predominant solid pattern can mimic ALK- ALCL. This tumor shows amphophilic to basophilic cytoplasm, marked anaplasia, and is strongly positive for CD30 (Figure 7B), cytokeratin, and germ cell transcription factors, and is negative for T-cell antigens, CD45, and CD43. Poorly differentiated carcinomas are positive for cytokeratins and negative for CD30, T-cell markers, and may or may not be positive for organ-specific transcription factors (p63, TTF1, PAX8, etc.). A careful interpretation of EMA should be performed when the differential diagnosis includes metastatic carcinoma and ALK- ALCL, since EMA may be positive in the latter. Rare cases of ALK- ALCL can be focally positive for cytokeratins and misdiagnosed as a poorly differentiated carcinoma, particularly if EMA is also positive [44,45,46,55] (Figure 5F, inset). Moreover, ALK- ALCL cases with the *TP63* rearrangement (see section on molecular alterations) are positive for p63 by immunohistochemistry [40,47] and the interpretation of the isolated expression of p63 does not always translate into a tumor with squamous cell differentiation.

##### ALK- ALCL vs. Other Epithelioid Malignancies

Although some of these tumors may be distinguished readily by morphology from ALCL by the lack of “hallmark” cells, some others may show overlapping morphology with this type of TCL. ALCL is negative for cytokeratins, vascular markers, melanoma markers, TFE3, and retains the expression of INI1/SMARCB1. On the other hand, non-hematopoietic epithelioid malignancies are positive for some of the markers mentioned above and are negative for T-cell markers, CD30, CD43, and CD45. For example, rhabdoid neoplasms show abundant glassy eosinophilic cytoplasm and show loss of INI1/SMARCB1. Epithelioid sarcoma may mimic ALCL, but this tumor is positive for vascular markers (CD34, ERG, FLI1), cytokeratin, and shows loss of INI1/SMARCB1 (Figure 7C). Alveolar soft part sarcoma demonstrates a nested and pseudoalveolar pattern not seen in ALCL but in some cases this distinction may be difficult, namely a core biopsy or material that has not been properly fixed. Alveolar soft part sarcoma is positive for TFE3. The very rare epithelioid myofibroblastic inflammatory sarcoma may resemble ALCL, but this tumor is positive for ALK (nuclear membrane or perinuclear pattern), desmin, CD30, and focally for SMA [62]. A metastatic melanoma may show epithelioid and anaplastic features, but this tumor is positive for S100, Melan-A, HMB-45, and SOX10 and only rarely shows expression of CD30 (Figure 7D).

##### Sarcomatoid ALK- ALCL vs. other Spindle Cell Tumors

The morphology of an inflammatory pseudotumor is diverse and some cases may show significant atypia to consider the possibility of a high-grade sarcoma, and for the purpose of this review also of sarcomatoid ALK- ALCL. An inflammatory pseudotumor can be positive or negative for ALK (~50% of cases), positive for SMA, and is negative for desmin, CD30, and T-cell markers (highlight background small T-cells). High grade spindle cell sarcomas may show a diverse expression of markers but are negative for CD30 and T-cell markers. However, immunohistochemistry for CD30 and T-cell markers is not typically performed as part of the diagnostic work up of sarcomas, resulting in the under recognition of sarcomatoid ALK- ALCL.

We strongly recommend performing CD43, CD30, and ALK in any poorly differentiated malignant neoplasm with epithelioid or spindle morphology particularly when multiple markers have failed to confirm a specific lineage (negative S100, CD45, and cytokeratins) to not miss the diagnosis of ALK- ALCL. This is particularly important for some ALCL variants as well as for cases with a “null” phenotype. If CD30 is positive, *TCR* clonality analysis may be an additional helpful test to further support a diagnosis of ALK- ALCL.

### 2.6. Genetic Findings and Molecular Alterations

About 70–90% of ALCL cases (ALK+ and ALK-) show clonal rearrangement of the *TCR* genes irrespective of the T-cell antigen status [41]. *TCR* clonality supports a diagnosis of ALK- ALCL in cases showing the appropriate morphology, the expression of CD30, and a “null” phenotype [41,63]. In one study, 20/31 (65%) of ALK- ALCL cases demonstrated gains of 1q and 6p21 by comparative genomic hybridization and these alterations were different from those found in ALK+ ALCL. However, none of these chromosomal abnormalities were associated with survival [64]. To the best of our knowledge, until now there is no recognized cytogenetic and/or molecular alteration that may explain the development of “hallmark” cells and/or “doughnut” cells in this lymphoma.

**Figure 8:**
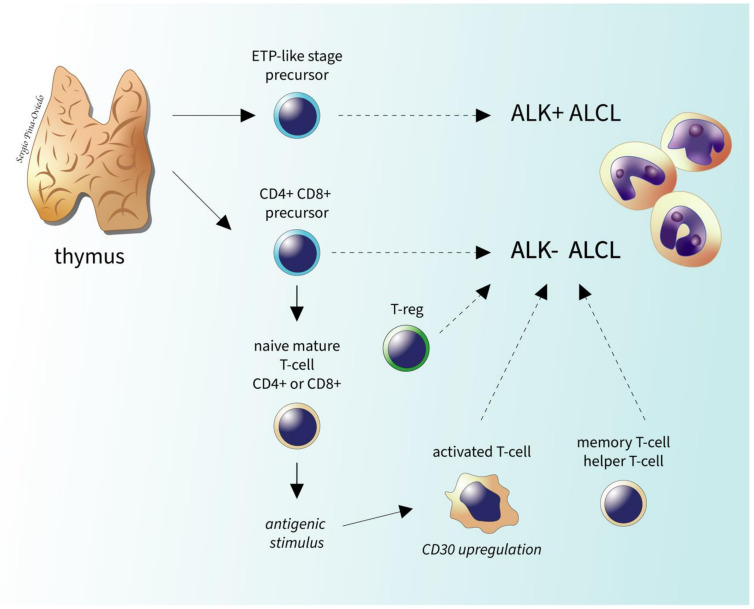
Putative cell(s) of origin of systemic ALCL. The methylation pattern of ALCL is similar to that of immature T-cells/thymocytes at certain stages of differentiation. ALK- ALCL has a methylation profile similar to a double-positive (CD4+/CD8+) thymic precursor. Nevertheless, multiple other subtypes of mature T-cells might also represent a potential precursor of this lymphoma (dashed arrows). ALK+ ALCL may also derive from similar mature T-cell subtypes as ALK- ALCL but this is not illustrated for simplicity.

Using DNA methylation profiling it has been discovered that ALK- and ALK+ ALCL have highly similar genome-wide DNA methylation in the genes involved in T-cell differentiation and immune responses (*TCR*, *CTLA-4*) and these methylation patterns resemble those of thymic progenitor cells, suggesting a possible thymic origin of ALCL: a double-positive CD4/CD8 precursor T-cell for ALK- ALCL and a CD34+/CD1a− precursor T-cell for ALK+ ALCL [65] (Figure 8).

#### 2.6.1. ALK- ALCL, STAT3, and JAK1 Mutations

**Figure 9:**
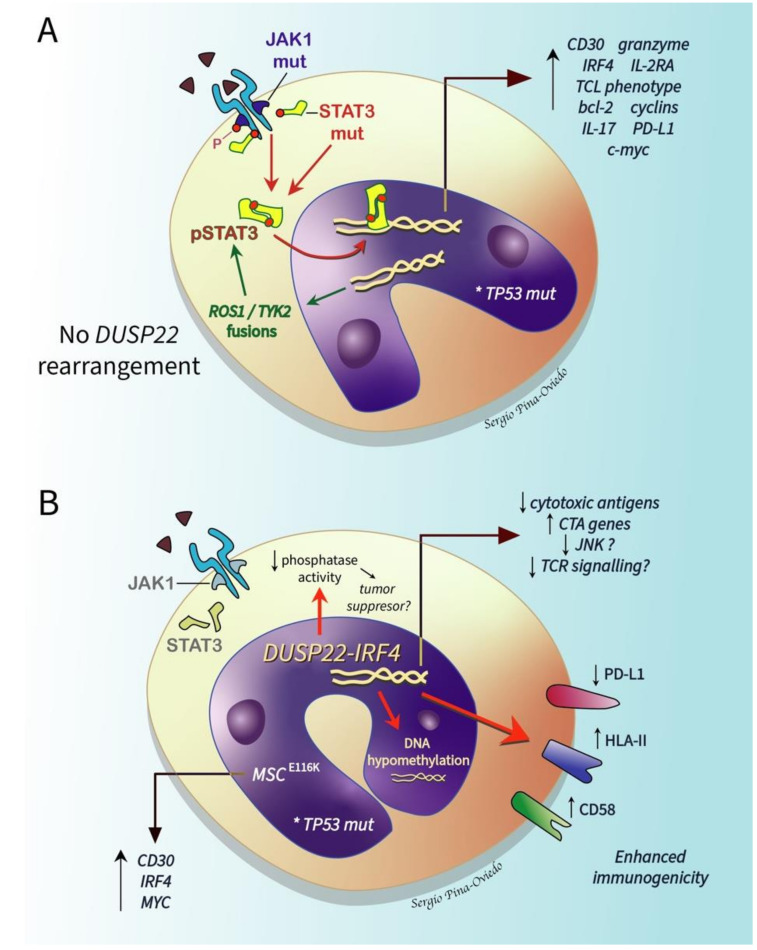
Molecular pathogenesis of systemic ALK- ALCL. (**A**) Mutations (mut) of JAK1 and/or STAT3 as well as the co-mutation of JAK and STAT have been implicated in the pathogenesis of this lymphoma. These mutations result in the constitutive activation of STAT (phosphorylated STAT; pSTAT3) that translocates to the nucleus and induces multiple genes resulting in the expression/activation of the molecules listed and of a T-cell lymphoma (TCL) phenotype Additionally, chimeric fusion genes with concomitant transcriptional and kinase activity (*ROS1*, *TYK2*) are also capable of sustaining the ALCL phenotype via pSTAT3. The activation of the JAK/STAT pathway is only found in cases without the *DUSP22* rearrangement. (**B**) ALK- ALCL with *DUSP22-IRF4* rearrangement/t(6;7)(p25.3;q32.3) is associated with downregulation of DUSP22 (dual specificity phosphatase 22) and likely its decreased phosphatase activity that may result in tumor suppressor effects that are yet unknown. The *DUSP22-IRF4* rearrangement has been associated with a decreased expression of cytotoxic molecules, marked DNA hypomethylation, and an enhanced immunogenicity via the overexpression of immunogenic cancer-testis antigen (CTA) genes, HLA class II and CD58, and the downregulation of PD-L1. These mechanisms may contribute to the favorable prognosis seen in this group of ALK- ALCLs. Most *DUSP22*-rearranged cases also harbor a recurrent mutation in musculin (*MSC^E116K^*) that induces the expression of the CD30–IRF4–MYC axis. In vitro studies have shown that DUSP22 has the capacity to suppress TCR-induced activation/signaling and JNK signaling that could also have an effect on the pathogenesis of this lymphoma. In both scenarios, the presence or acquisition of a *TP53* mutation (*) confers a bad prognosis.

Even though ALK- ALCL pathogenesis is unrelated to ALK activation, ALK+ and ALK- ALCLs share a STAT3-mediated oncogenic mechanism. Two mutually exclusive mechanisms independent of ALK activation lead to the constitutive activation of STAT3 in ALK- ALCL: (1) oncogenic point mutations in *JAK1* and/or *STAT3* (~20% of cases), and (2) oncogenic fusion genes displaying concomitant transcriptional and kinase activities capable of sustaining the ALCL phenotype via STAT3, namely *NFKB2-ROS1*, *NCOR2-ROS1*, *NFKB2-TYK2*, and *PABPC4-TYK2* fusions (rare cases) [66] (Figure 9A). Therefore, it is possible that JAK/STAT3 pathway inhibitors may have a therapeutic application not only in ALK+ ALCL, but also in ALK- ALCL [66].

A recent multi-institutional study using deep targeted next generation sequencing of 47 ALK+ and 35 ALK- ALCLs has identified that ALK- ALCLs have an average of 4.2 mutations per patient, whereas ALK+ cases have an average of 2.6 mutations per patient [67]. Mutated genes predictive of a poor prognosis (defined as death of disease, refractory to therapy, and/or relapsed disease) independent of ALK status included *TP53*, *STAT3*, *EPHA5*, *JAK1*, *PRDM1*, *LRP1B*, and *KMT2D.* From all these, the *STAT3* and *JAK1* mutations were identified in 26% of ALK- ALCLs and the *STAT3* and *TP53* mutations had shorter overall survivals [67]. Similarly, the expression of p53 and beta-catenin, and the co-expression of these markers at the protein level has been observed more in association with ALK- ALCL than with ALK+ ALCL and it appears to correlate with a poor prognosis [68].

In 2014 it was identified that 30% of ALK- ALCLs show a rearrangement of *DUSP22* (dual-specificity phosphatase 22), 8% of cases show a rearrangement of *TP63*, and the rest of cases lack these rearrangements [40]. Both the *DUSP22* and *TP63* gene rearrangements are detectable by FISH, are mutually exclusive, and correlate strongly with prognosis. Therefore, ALK- ALCL is now subclassified into *DUSP22*-rearranged, *TP63*-rearranged, and triple negative for *ALK*, *DUSP22*, and *TP63* rearrangements. *DUSP22*-rearranged cases have a similar 5-year overall survival as ALK+ ALCLs (85–90%), triple negative cases have intermediate prognosis (42%, 5-year overall survival), and cases with the *TP63* rearrangement have a poor prognosis (17%, 5-year overall survival) [40]. FISH to evaluate for these genetic alterations is still not widely available but it is now becoming more widespread. *DUPS22* and *TP63* gene rearrangements are absent in ALK+ ALCL but can be found in a subset of PTCL, NOS, and therefore, they support but do not confirm the diagnosis of ALK- ALCL and should be interpreted in the right context [53]. The prognostic impact of these rearrangements is also likely affected by the presence/absence of *STAT3* and *TP53* mutations, as recently shown by a next generation sequencing study in a large cohort of patients with ALCL [67].

#### 2.6.2. DUSP22-Rearranged ALK- ALCL

About 30% of ALK- ALCLs show a rearrangement of *DUSP22* that occurs near the *DUSP22-IRF4* locus on 6p25.3. The *FRA7H* fragile site on 7q32.3 is the most common partner gene resulting in the translocation t(6;7)(p25.3;q32.3). This translocation is associated with downregulation of DUSP22 and upregulation of *MIR29* microRNAs on 7q32.3 but with no alteration in the expression of *IRF4* [69]. This subgroup represents a distinct subset of ALK- ALCL because these cases (1) lack the expression of the genes associated with JAK-STAT3 signaling, (2) overexpress immunogenic cancer-testis antigen genes, (3) show marked DNA hypomethylation, (4) have a markedly reduced expression of PD-L1 and a high expression of CD58 and HLA class II [70] (Figure 9B). These differences likely contribute to the favorable prognosis of this group. Pharmacologic DNA demethylation of ALCL cell lines without *DUSP22* rearrangement recapitulates the overexpression of cancer-testing antigens and other *DUSP22* signature genes, suggesting that demethylating agents might reprogram aggressive subtypes of ALK- ALCL into a “*DUSP22*-like immunogenic molecular signature” with a potential impact on prognosis [70]. A novel recurrent mutation in the musculin gene, *MSC^E116K^*, that induces the expression of the CD30–IRF4–MYC axis and drives cycle cell progression has been recently reported in ALK- ALCL, nearly exclusively in the *DUSP22*-rearranged cases [71] (Figure 9B).

Dual specificity phosphatases (DUSPs) are a large family of proteins that dephosphorylate phosphotyrosine and phosphoserine/phosphothreonine residues on several molecules [72]. DUSPs are expressed in multiple tissues and some of them are differentially expressed in resting and activating immune cells. DUSPs are divided into several groups: slingshots, phosphatases of regenerating liver, cell division cycle (Cdc14) phosphatases, phosphatase and tensin homologues deleted on chromosome 10 (PTEN), myotubularins, mitogen-activated protein kinase phosphatases (MKPs), and atypical DUSPs [72]. They have multiple functions, including the major modulation of critical cellular signaling pathways. DUSP22 was discovered in 2001 and belongs to the group of atypical DUSPs that lack the kinase interaction motif (KIM) found in classic DUSPs and in MKPs [73]. DUSP22 may have ≥1 physiological substrate as well as multiple functions that may be lineage specific. Myeloid cells have the highest levels of DUSP22 mRNA, whereas the heart and the muscles have the highest protein expression in mice tissues [74]. In vitro studies have shown that DUSP22 has the capacity to suppress TCR-induced activation/signaling and JNK signaling, hence its alternative name “JNK pathway-associated phosphatase” (JKAP) [75,76]. DUSP22 also has a role in the inhibition of interleukin-6 (IL-6)-induced STAT3 activation; the silencing of DUSP22 using siRNA enhances the phosphorylation and activation of STAT3 in cell lines [77]. DUSP22 appears to play an important role in autoimmune disease in humans and mice models. Its expression is inversely correlated with disease activity in inflammatory bowel disease, and its downregulation in CD4+ T-cells correlates with the activity of systemic lupus erythematosus [78,79]. The reduced expression of DUSP22 has been identified in TCL and this phosphatase likely behaves as a tumor suppressor gene in ALK- ALCL [53,70].

#### 2.6.3. TP63-Rearranged ALK- ALCL

Eight percent of ALK- ALCLs show a rearrangement of *TP63* in 3q28, most commonly with *TBL1XR1* as a result of inv(3)(q26q28) [40]. As mentioned above, this subgroup has the worst prognosis of all ALK- ALCLs, but since these cases are rare, it is difficult to determine further specific histopathologic features. Morphologically, these cases tend to have a lower amount of large pleomorphic cells similar to what is seen in *DUSP22*-rearranged cases [35].

p63 is a member of the p53 family that was originally described in 1997–1998. The *TP63* gene is expressed either as a full-length isoform containing the transactivator domain (TAp63) or as an amino-deleted isoform (ΔNp63) [80]. p63 has multiple effects in cancer, namely the regulation of cell cycle arrest, apoptosis, stemness, and tumorigenesis [81]. p63 has been detected in multiple solid tumors, particularly carcinomas, and few studies have evaluated its expression in lymphomas. TAp63 is the most common expressed isoform in B-cell lymphomas, best known in primary mediastinal (thymic) large B-cell lymphoma [82,83,84,85,86,87,88].

The largest study on p63 protein expression and correlation with *TP63* abnormalities in ALCL (116 cases) demonstrated that p63 was positive by immunohistochemistry in ~33% of ALK- ALCLs, ~31% of pc-ALCLs, and in 5% of ALK+ ALCLs [47]. The higher frequency of p63 protein expression compared to that of *TP63* rearrangement (33% vs. 8%) is explained by the presence of extra copies of *TP63* in cases without rearrangement. TAp63 is the isoform expressed in all ALCLs whereas ΔNp63 (i.e., p40) is not. Immunohistochemistry for p63 is not specific for a *TP63* rearrangement but it is a useful screening test to select cases of ALK- ALCL for *TP63* FISH, particularly in cases with >30% positive lymphoma cells [47]. The biological significance of p63 expression and *TP63* genetic alterations in ALK- ALCL remains unknown.

#### 2.6.4. Genetic Abnormalities with a Potential Impact on the Differential Diagnosis of ALK- ALCL vs. CD30+ Large Cell PTCL, NOS

There have been multiple studies that have tried to determine the pathogenetic differences between ALK- ALCL and PTCL, NOS. Using RT-qPCR, a study from the European T-cell lymphoma group validated a three-gene model (*TNFRSF8*, *BATF3*, *TMOD1*) able to separate ALK- ALCL from PTCL, NOS. When these three genes are expressed, ALK- ALCL could be distinguished from PTCL, NOS with an accuracy of ~97% [89]. The most overexpressed genes in ALK- ALCL in this study were *CD80*, *DC86*, *CCND2*, and *MIR155HG*. Other studies have demonstrated the overexpression of *CCR7*, *CNTFR*, *IL22*, and *IL21* [90].

*JAK2* rearrangements have been recently identified in cases of CD30+/ALK- PTCL with an anaplastic morphology and eosinophilia that show CHL-like features and CD15 expression [91], suggesting that this abnormality might help to distinguish between PTCL with CHL-like features and ALK- ALCL with a “Hodgkin-like” pattern. Further studies are needed to support these findings. Lastly, about 25% of ALK- ALCLs express an oncogenic truncated Erb-B2 receptor tyrosine kinase 4 (*ERBB4*) that is not detected in ALK+ ALCL and PTCL-NOS [92]. These cases also show a “Hodgkin-like” pattern. *ERBB4* expression appears to be mutually exclusive of *DUSP22*, *TP63*, *ROS1*, and *TYK2* rearrangements and could potentially become another subgroup of ALK- ALCL [92].

**Figure 10:**
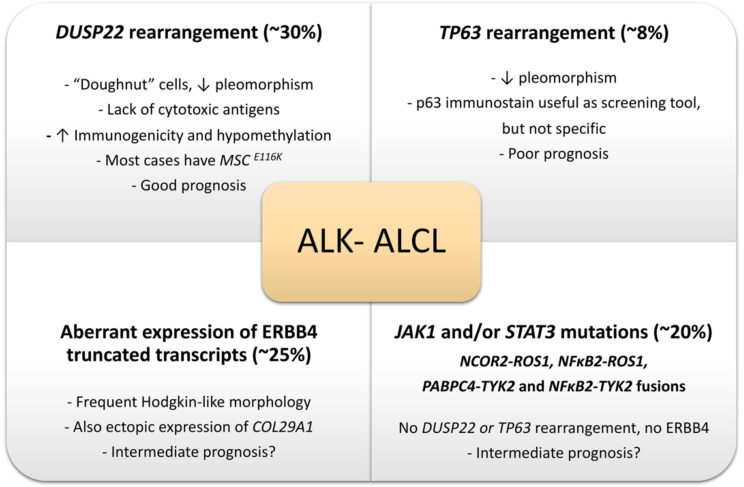
Relevant molecular alterations in systemic ALK- ALCL (see also text).

The relevant molecular alterations known to date in ALK- ALCL are summarized in Figure 10.

## 3. Primary Cutaneous ALCL

### 3.1. Introduction

According to the 2016 definitions of the WHO and the European Organization for Research and Treatment of Cancer (EORTC), primary cutaneous CD30 lymphoproliferative disorders include pc-ALCL, lymphomatoid papulosis (LyP), and borderline lesions [93,94]. They represent 30% of cutaneous TCLs and are the second most common group of cutaneous TCLs after mycosis fungoides [93]. LyP and pc-ALCL share medium to large CD30+ atypical lymphoid infiltrates but represent two ends of a spectrum of diseases that have many overlapping histological and immunohistochemical features. However, they have different clinical presentations, clinical courses, and prognoses. As a consequence, the diagnosis should be based on the correlation of clinical, histopathological, phenotypic, and genetic features, as well as the staging results [95].

### 3.2. Historical Aspects and Definition

In 1989, Berti et al. of the University of Milan, Italy, described pc-ALCL in a case that was misdiagnosed as cutaneous metastasis [96]. Pc-ALCL is a rare form of ALCL that has no systemic involvement at the time of diagnosis and in the following six months [97]. Pc-ALCL is a TCL that is distinguished by the presence of large lymphoid cells with marked nuclear pleomorphism and expression of CD30. It is characterized by an indolent course, often with rapid development that may simulate aggressive lymphomas. However, it shows a favorable prognosis with a 10-year overall survival rate of 90% [98]. Extra-cutaneous spread occurs in 10% of cases and it is most likely to affect regional lymph nodes. Pc-ALCL should be distinguished from systemic ALCL with cutaneous involvement as a separate disease with distinct clinical and cytogenetic features, as well as different outcomes (see prior section) [94,99].

### 3.3. Epidemiology, Risk Factors, and Clinical Features

Pc-ALCL accounts for ~9% of all cutaneous TCLs, and the age of presentation is around 60 years. It predominantly affects males, and it is more common in the Caucasian population [100]. This cutaneous TCL has rarely been described in children, and at least one congenital case has been documented [101,102]. Pc-ALCL is a common form of cutaneous lymphoma in immunosuppressed patients, including those infected with human immunodeficiency virus and organ transplant recipients [103].

**Figure 11:**
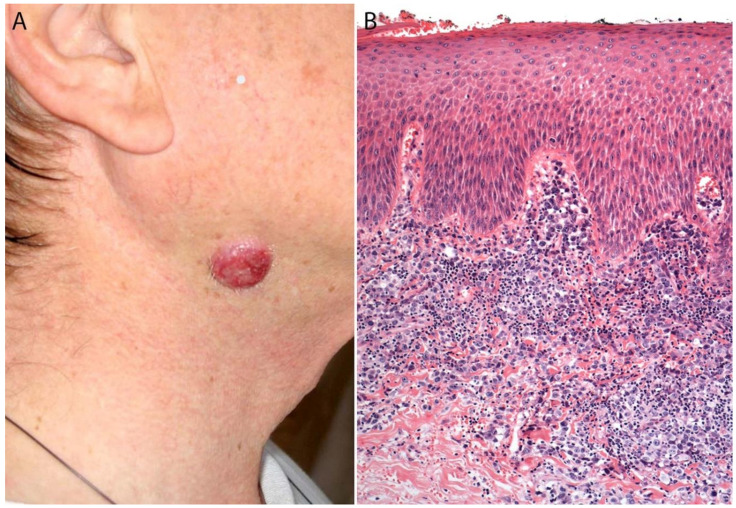
(**A**) Primary cutaneous ALCL. A 45-year-old man presented with a single cutaneous erythematous nodule located in the neck. (**B**) Microscopically, the nodule consists of a non-epidermotropic atypical lymphoid infiltrate with diffuse dermal involvement.

Clinically, pc-ALCL most frequently affects the trunk, face, and extremities (Figure 11A). Most patients present with large (>2 cm) nodules or papules that grow rapidly. They can be single or sometimes grouped and are often ulcerated and reddish-brown. Although pc-ALCL may sometimes undergo spontaneous regression in ~25% of patients, it is less likely to self-resolve than LyP [94,97]. Relapse is common and progression to systemic disease is rare [104]. Patients with extensive skin lesions on the legs or arms show statistically significant worse prognoses [103].

Pc-ALCL may be induced in patients receiving various immunomodulatory drugs, such as the tumor necrosis factor (TNF) blocker adalimumab used to treat various autoimmune diseases, and fingolimod, used to treat patients with relapsing-remitting multiple sclerosis [105,106]. Most cases of pc-ALCL can be effectively managed with radical excision or radiation when presenting with an isolated lesion. However, some patients will experience relapse and require additional therapy [107].

### 3.4. Pathology

**Figure 12:**
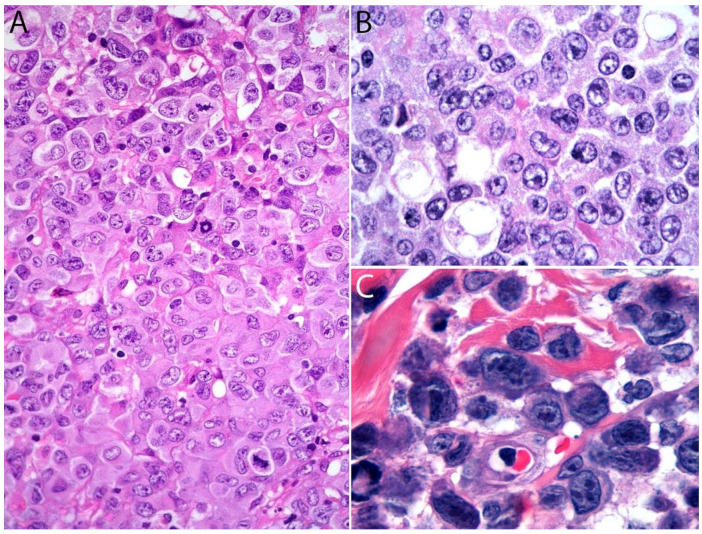
(**A**–**C**). Primary cutaneous ALCL is composed of sheets of medium to large neoplastic lymphoid cells with anaplastic features.

**Figure 13:**
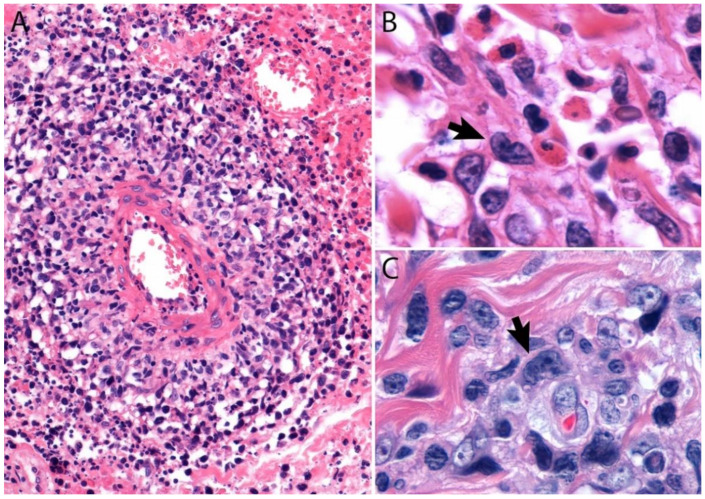
Primary cutaneous ALCL. (**A**) Diffuse atypical lymphoid infiltrate with pleomorphic cells with angiocentric growth and focal dermal necrosis. (**B**,**C**) Many cells have eccentric, kidney-shaped, or lobulated nuclei with small-to-medium size nucleoli and abundant eosinophilic cytoplasm, also known as “hallmark” cells (arrows).

**Figure 14:**
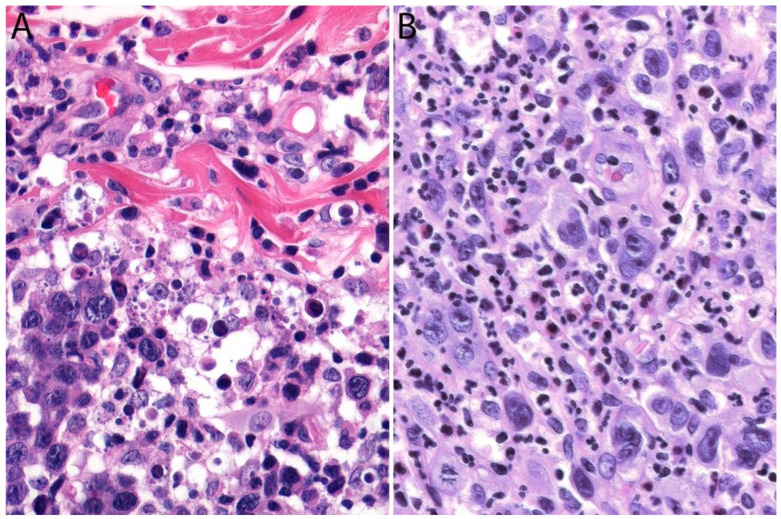
(**A**) Primary cutaneous ALCL with pleomorphic/anaplastic cells with numerous apoptotic bodies. (**B**) Extensive neutrophilic infiltrate in a case of “neutrophil-rich” pc-ALCL (“pyogenic cutaneous lymphoma”).

Histologically, pc-ALCL is characterized by a diffuse dermal infiltrate composed of cohesive sheaths of large anaplastic, pleomorphic, or immunoblastic cells with abundant cytoplasm. Epidermotropism is usually absent or subtle [98,108] (Figure 11B and Figure 12). In addition to a sheet-like pattern of growth, the neoplastic infiltrate has a striking angiocentric disposition and subcutaneous involvement can be prominent (Figure 13A). “Hallmark” cells and “doughnut” cells are also present [34,109] (Figure 13B,C). Apoptotic bodies may be numerous, and reactive B- and T-cells are often present at the periphery of the lesion (Figure 14A).

It is important to note that a non-anaplastic appearance may be seen in 20% of cases of pc-ALCL and there are no differences in clinical behavior between the anaplastic and non-anaplastic variants [110]. A “small cell variant” composed of small to medium-sized hyperchromatic atypical lymphocytes that occurs almost exclusively in middle-age or elderly men was recently described [111]. Reed-Sternberg-like cells may often be present, and although not common, an inflammatory background of reactive T-cells, eosinophils, and neutrophils may be as prominent as seen in LyP. A rare neutrophilic-rich variant called “pyogenic cutaneous lymphoma” is associated with pseudocarcinomatous hyperplasia and is more commonly seen in immunosuppressed patients [112] (Figure 14B). This variant is characterized by ulceration and the formation of small abscesses within the tumor. Neutrophils are attracted via IL-8 production by the CD30+ tumor cells and IL-8 levels are increased in cultured tumor cells and in the serum of affected individuals [113]. Caution must be taken when examining a case of this variant because it may be confused with various infectious and non-infectious diseases, including Sweet syndrome, pyoderma gangrenosum, pyoderma faciale, and deep fungal infections [112,113,114].

In 2013, Kempf et al. described a rare variant of pc-ALCL with marked angiocentricity and angiodestruction resembling angioinvasive LyP type E [115]. It cannot be completely excluded that angiocentric pc-ALCL may precede angiocentric-type E LyP in some patients, which further supports the concept that LyP and pc-ALCL are part of a clinicopathological spectrum [115]. Due to the angiodestructive pattern of this variant, the differential diagnosis includes extranodal NK/T-cell lymphoma, nasal type; adult T-cell leukemia/lymphoma; cutaneous γ/δ+ TCL; and EBV-associated *hydroa vacciniforme*-like lymphoproliferative disorders [115].

Researchers from the Hospital Universitario Marqués de Valdecilla in Santander, Spain, described cases of pc-ALCL harboring translocations involving the *DUSP22-IRF4* locus [116]. This variant is characterized by a striking biphasic histopathological pattern composed of medium to larger transformed CD30+ lymphocytes with abundant finely granular cytoplasm, numerous mitoses and apoptotic bodies, “hallmark” cells diffusely infiltrating the dermis, smaller atypical CD30+ lymphoid cells infiltrating the epidermis, and a “pagetoid reticulosis-like” pattern occasionally forming “Pautrier-like” microabscesses [116,117]. Various other unusual morphologic variants have been described with no prognostic significance, including cases with intratumoral vascular and lymphatic vessel involvement [118], intra-lymphatic location [119], angiodestructive forms [115], pseudoepitheliomatous hyperplasia/keratoacanthoma-like features [120], myxoid stroma, or prominent spindle cell/sarcomatoid morphology [121,122]. Pseudoepitheliomatous hyperplasia may occur in up to 20–30% of cases but it is not exclusive of pc-ALCL since this phenomenon has also been reported in patients with LyP [115].

#### Immunohistochemistry

**Figure 15:**
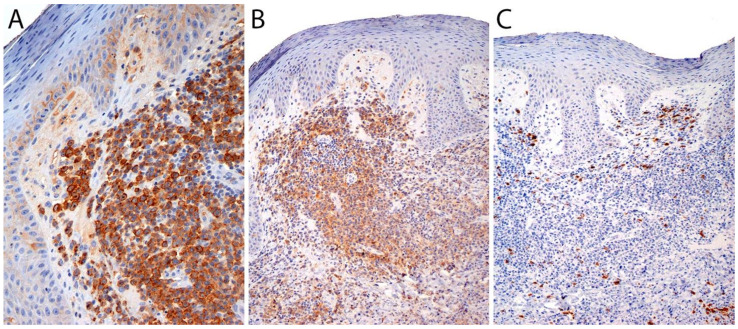
Immunohistochemical findings in primary cutaneous ALCL. The tumor cells are diffusely positive for (**A**) CD30 with a membranous and Golgi (dot-like) pattern. The tumor cells are positive for (**B**) CD4, and negative for (**C**) CD8.

The neoplastic cells in pc-ALCL show an identical immunophenotype to that of systemic ALK- ALCL. CD30 is expressed in >75% of neoplastic cells (Figure 15). Most cases are positive for CD45, usually positive for CD4, and show a variable expression of other T-cell markers, including CD2, CD5, CD7, and CD45RO (Figure 15). A negative or dim expression of CD3 is secondary to genetic alterations in *TCR* coding regions on chromosome 1 [123]. Up to 31% of cases may show loss of at least two T-cell antigens. In addition, cytotoxic features are revealed by the expression of granzyme B, TIA-1, and perforin. Roughly 18% of cases can be positive for CD8, and 26% of cases are negative for both CD4 and CD8, which is more common in systemic ALCL than in pc-ALCL [63]. It is worth noting that CD30 is found at variable levels in different lymphomas of B-cell or T-cell derivation, myeloblasts in a subset of patients with acute myeloid leukemia, in myelodysplastic syndrome, and in normal plasma cells and activated macrophages [124]. If the tumor cells show weak or partial CD30 expression, it should raise concern for cutaneous involvement by another subtype of TCL [97]. CD30 can also be detected in activated B- and T-cells in non-neoplastic cutaneous infiltrates, as seen in cases of arthropod bites (tick, scabies, *Leishmania*), various infections (syphilis, herpes simplex virus, varicella zoster virus, molluscum contagiosum), hidradenitis, ruptured cysts, rhinophyma, certain drug eruptions, and stasis ulcers. CD43 is positive in most cases, whereas EMA is less often expressed than in systemic ALCL. In fact, a strong and diffuse expression of EMA should suggest cutaneous involvement of systemic ALCL [63].

The transcription factor GATA3 is negative or weakly positive in pc-ALCL, which is a useful finding in distinguishing it from CD30+ mycosis fungoides with large cell transformation that are typically strongly and diffusely positive for GATA3 [125]. Studies by Mitteldorf et al. have shown that galectin 3 (Gal-3), a β-galactoside-binding protein, is expressed in pc-ALCL in contrast to its lower expression in mycosis fungoides with large cell transformation. Therefore, it represents an additional tool to differentiate these entities [126].

Clusterin is a ubiquitous 80-kDa heterodimeric glycoprotein that is expressed in pc-ALCL with a distinct Golgi (dot-like) pattern [127]. Clusterin does not reliably distinguish between LyP, pc-ALCL, and mycoses fungoides in large cell transformation, but it may be useful to discern pc-ALCL from some reactive cutaneous inflammatory conditions [127]. The expression of CD71 (transferring receptor-1), HLA-DR, and CD25 (alpha chain of the IL-2 receptor) has been demonstrated in approximately half of the cases [103].

Immunostaining for the ALK protein is probably the most helpful feature in differentiating between pc-ALCL and systemic ALK+ ALCL. About 50–60% of systemic ALCL cases harbor a translocation t(2;5) involving the *ALK* gene, but it has been reported only rarely in cases presenting primarily in the skin [123]. The immunohistochemical evaluation of ALK is typically negative in pc-ALCL, but in rare cases of the disease limited to the skin, ALK expression has been documented, particularly in children [128,129,130]. In contrast to systemic ALCL, cases of pc-ALCL that show positive ALK immunostaining seem to have a favorable outcome comparable to that of patients with ALK- pc-ALCL. The morphological features of the lymphoma cells are not sufficiently different to distinguish ALK+ and ALK- cases based on hematoxylin and eosin staining.

Up to 50% of the neoplastic cells in pc-ALCL express the cutaneous lymphocyte antigen (CLA) [131], whereas this antigen is negative in systemic ALCL. CD15 may be positive in up to 40% of cases, which makes it difficult to differentiate from CHL. In pc-ALCL, however, large cells predominate and mainly resemble immunoblasts, “hallmark” cells are frequent, and only occasionally are Reed-Sternberg-like cells present. Unlike CHL, PAX5 is rarely expressed in pc-ALCL, but the transcriptional factor MUM1/IRF4 is positive in most cases of pc-ALCL [94,132].

Although CD56 is generally negative in pc-ALCL, Yu et al. found that 2/148 cases (1.4%) were positive for CD56 [133]. EBV is negative in pc-ALCL either in LMP-1 immunohistochemistry or EBER in situ hybridization. Therefore, EBV positivity should raise concern for cutaneous involvement by CHL or other lymphomas, such as extranodal NK/T-cell lymphoma, nasal type, or B-cell lymphoma with plasmablastic differentiation [97].

### 3.5. Differential Diagnosis

The differential diagnosis of pc-ALCL is often challenging and includes a broad range of primary cutaneous and systemic large-cell lymphomas [94,95,98,123]. There is a significant histological overlap between LyP types A and C and pc-ALCL, and to date, no reliable biomarker has been found to distinguish between these entities. For this reason, correlation between the clinical, histological, and immunohistochemical features is needed to avoid making a wrong diagnosis [108].

Several other neoplasms are also included in the differential diagnosis of these CD30+ lymphoproliferative disorders, including systemic ALCL with cutaneous involvement as well as mycosis fungoides with large cell transformation. A careful review of the clinical, histological, and immunohistochemical features is useful in differentiating these conditions. Systemic ALCL has a very different clinical course and a much poorer prognosis than pc-ALCL.

The main differential diagnoses of pc-ALCL are summarized in Table 3.

### 3.6. Genetic Findings and Molecular Alterations

Most cases of pc-ALCL (65–90%) demonstrate a clonal *TCR* gene rearrangement but lack the expression of TCR proteins [94,134]. Array-based comparative genomic hybridization studies have revealed chromosomal imbalances in ~40% of cases in chromosomal regions coding for *FGFR1* (8p11), *NRAS* (1p13.2), *MYCN* (2p24.1), *RAF1* (3p25), *CTSB* (8p22), *FES* (15q26.1), and *CBFA2* (21q22.3) [135]. Gains on chromosomes 7q31 and 17q and losses in the 6q16–6q21, 6q27, and 13q34 regions have also been detected [136]. Pc-ALCL shows a higher expression of the skin-homing chemokine receptor genes *CCR10* and *CCR8* and aberrant expression of genes implicated in apoptosis (*TNFRSF8/CD30*, *TRAF1*) and proliferation (*IRF4/MUM1*, *PRKCQ*) [94,103,135].

In systemic ALK- ALCL and pc-ALCL, t(6;7)(p25.3;q32.3)(*DUSP22/FRA7H)* was originally reported in up to 10% of cases [69]. Additional cases with rearrangements of 6p25.3 not involving 7q32.3 have also been reported [69]. In 2013, Karai et al. used a FISH analysis to describe a rearrangement of *IRF4* and *DUSP22* on the 6p25.3 locus, a tumor-suppressor gene that regulates T-cell signaling and proliferation in LyP and pc-ALCL [137]. The 6p25.3 rearrangement represents genetic evidence of a continuous spectrum between these two entities. Rearrangements of 6p25.3 are absent in conventional mycosis fungoides and mycosis fungoides with large cell transformation, and therefore, they represent a useful tool to distinguish these entities from pc-ALCL [137].

The majority of pc-ALCLs are triple negative for rearrangements in *ALK*, *DUSP22*, and *TP63*. However, the *DUSP22* rearrangement has been described in ~25% of cases [47,116]. Patients with pc-ALCL do not carry *ALK* translocations and are negative for ALK by immunohistochemistry. However, rare cases of pc-ALCL may show strong nuclear and cytoplasmic or only cytoplasmic ALK immunostaining [138].

The genetic and molecular alterations in pc-ALCL are summarized in Table 4.

## 4. Breast Implant-Associated ALCL

### 4.1. Definition

BIA-ALCL is a relatively newly recognized clinicopathological entity with a unique morphologic pattern. It is defined as a TCL with morphological and immunophenotypic characteristics that are indistinguishable from ALK- ALCL that present as an effusion formed around a breast implant and the fibrous capsule. BIA-ALCL may spread to the adjacent breast parenchyma, regional lymph nodes, or more rarely, it may spread systemically. BIA-ALCL is currently included as a provisional entity in the revised 4th edition of the WHO classification of tumors of hematopoietic and lymphoid tissues (2017) [139] and in the 5th edition of the WHO classification of breast tumors (2019) [140].

### 4.2. Historical Aspects

The first case of BIA-ALCL was described by Keech and Creech in 1997 [141]. Since then, there have been over 900 cases reported in the literature, most of which have had an indolent clinical behavior [142]. In 2008, Roden et al. suggested an increased risk of breast ALCL in patients with breast implants [143]. In 2014, Miranda et al. from M.D. Anderson Cancer Center in Houston, TX, US, published a study that described a group of 60 patients with BIA-ALCL and correlated the morphologic and immunophenotypic features with the clinical behavior [144].

### 4.3. Epidemiology, Risk Factors, and Clinical Features

Non-Hodgkin lymphoma (NHL) of the breast is rare. It accounts for <1% of malignant tumors at this site and ~2% of all extranodal lymphomas. The most common type of breast lymphoma is DLBCL followed by marginal zone lymphoma [145]. TCLs of the breast are even rarer. In a study of 106 breast lymphomas, only six cases of ALCL were identified, and two of these cases were associated with breast implants [146].

BIA-ALCL occurs more frequently in the U.S., Europe, and Australia as compared to Asia and Latin America. Over 550,000 breast implant surgeries are performed each year in the U.S. and ~5% of American women have undergone this procedure [147]. The frequency and risk of developing BIA-ALCL depends on (1) the type of implant, (2) the implant manufacturer, and (3) the time elapsed since implantation regardless of whether the implant was placed for cosmetic or reconstructive purposes [148]. Globally, the frequency of BIA-ALCL is 1 in 30,000 women with breast implants. The average latency time from implantation to the development of BIA-ALCL is 10 years, with a range from 2.2 months to 28 years [149]. The incidence of this lymphoma is difficult to calculate. In the U.S. its incidence has been estimated at 1 in 6120 women with breast implants per year, and 1 in 4120 women with textured breast implants per year, with a cumulative incidence of 3.31 per 1000 breast implants between 14 and 16 years after surgery [150].

The median age at diagnosis of BIA-ALCL is 52 years and most patients are females. However, sporadic reports have also been described in transgender women, all of them associated with breast implants [151]. In about two-thirds of the patients the main symptom is a late effusion, defined as the accumulation of fluid (improperly referred to as “seroma”) in the peri-implant/intracapsular space occurring at least 1 year after breast implant placement [30]. About 9 to 13% of late effusions seen in patients with breast implants are secondary to BIA-ALCL. Less common symptoms include skin erythema, capsular contracture, breast edema, a breast mass, or more rarely, regional lymphadenopathy [152,153].

Mammography is not a reliable imaging method to detect BIA-ALCL. A peri-implant effusion is easily detected by breast ultrasound, a technique with a higher sensitivity and specificity that also allows for the evaluation of implant integrity and the presence of a mass [154]. In cases of inconclusive ultrasound results, magnetic resonance imaging becomes an excellent tool for the detection of a mass and/or effusion. Positron emission tomography and computed tomography scans are useful for staging [154,155].

The clinical behavior of BIA-ALCL is generally indolent and depends on its clinical stage (see below). This lymphoma is confined to the capsule in >80% of cases [30]. Two separate groups of investigators studied the clinical behavior of BIA-ALCL and agreed with their conclusions [156,157]. One of these groups suggested dividing the disease into two clinicopathological variants, each with its own prognosis [157]. The one with the higher prevalence is defined as a proliferation of neoplastic cells confined to the fibrous capsule (or up to T3) that has an indolent clinical course and usually remains disease-free after the removal of the implant with its capsule [157]. The other variant, known as the infiltrative variant, shows a massive infiltration of the lymphoma cells into the adjacent tissues and may feature eosinophils and fibrosis resembling CHL. This variant usually behaves more aggressively and almost always requires complementary therapy in addition to implant removal.

Lymph node involvement has a prognostic impact on the evolution of BIA-ALCL since it represents disease progression or clinically aggressive disease at presentation. It has a 75% survival rate compared to 97.9% for cases without nodal involvement [156].

### 4.4. Pathophysiology

Although the pathophysiology of BIA-ALCL is not fully understood, it appears to be intimately related to the host’s inflammatory response to the implant, as well as to the type of implant shell. A high proportion of cases are associated with textured implants as opposed to smooth implants. It is the implant shell that is directly immunogenic, rather than its content, whether it is silicone gel or saline solution. One hypothesis suggests that particles from textured implant shells cause persistent antigenic stimulation, leading to the proliferation of macrophages and T-cells, and causing immune dysregulation in genetically susceptible individuals [158,159]. Initially, there is suppression of T regulatory cells secondary to secretion of IL-6 and other cytokines (IL-8, IL-17, TGF-β1, INF-γ) by macrophages, polymorphonuclear cells, and Th17 cells. This, in turn, causes fibroblast activation and proliferation, eventually leading to the formation of a connective tissue capsule around the implant [160,161]. If this inflammatory process is sustained, it results in the oligo and monoclonal proliferation of CD30+ activated T-cells that are intimately involved in the inflammatory positive feedback loop by releasing pro-inflammatory cytokines [162]. A similar mechanism has been suggested in the lymphoproliferative processes at other sites, particularly those associated with orthopedic, maxillofacial, and heart valve prostheses [163]. A possible contributing factor to the development of BIA-ALCL with textured implants is that there is a greater surface of contact with the surrounding breast tissue [164]. Another hypothesis is that the inflammation caused by breast implants is enhanced by lipopolysaccharides from bacteria, namely *Ralstonia* spp. or *Staphylococcus epidermidis*, that grow on the implant surface and produce a biofilm capable of causing an infection that perpetuates an environment of chronic inflammation resulting in chronic T helper 1 cell stimulation [165]. The colonization of texture implants—with a high surface area, as mentioned above—facilitates the production of this biofilm and when a certain level of bacterial load is surpassed, it may produce chronic antigen stimulation in genetically susceptible subjects [165,166,167]. This persistent inflammatory process may lead to the development of CD30+/ALK- T-cell clones that may eventually progress to BIA-ALCL after several (8–12) years [165,166,167]. It is very likely that the pathogenesis of BIA-ALCL depends on the combination of some—if not all—of these mechanisms (multifactorial) and some others that are yet unknown.

### 4.5. Handling of Cytology Material and Capsulectomy Specimens of BIA-ALCL

Periprosthetic fluid is usually cloudy and is obtained by fine-needle aspiration, preferably guided by ultrasound. The minimum volume required for analysis is 10 mL, but the ideal volume should be around 50 mL. This amount of fluid should provide sufficient material for cytologic evaluation and the preparation of cell blocks for immunostaining. If any additional or left-over fluid is available, it can be submitted for flow cytometry analysis [168,169]. Once a cytologic diagnosis of ALK- ALCL is established, an *en bloc* resection of the implant and the periprosthetic capsule is recommended.

**Figure 16:**
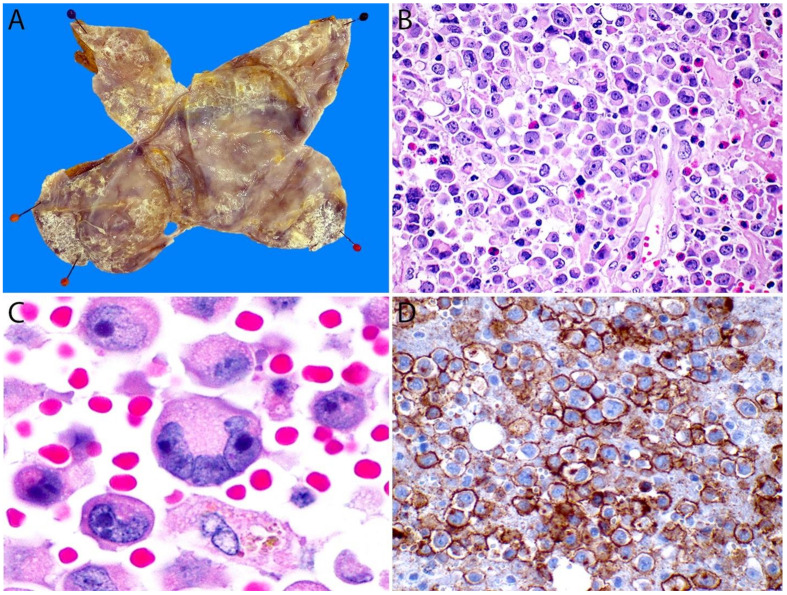
Breast implant-associated ALCL. (**A**) Opened breast implant capsule pinned to a flat surface (after overnight fixation). Note the multiple areas with a fibrinous/necrotic appearance on the luminal surface that correspond to collections of lymphoma cells. (**B**) The cell block obtained from the effusion contains numerous anaplastic large lymphoma cells. (**C**) At high magnification, the large lymphoma cells show a pleomorphic and hypechromatic nucleus with abundant eosinophilic cytoplasm. A “hallmark” cell is also seen (bottom). (**D**) The CD30 immunostain shows membranous and paranuclear dot (Golgi) labelling in virtually all these cells.

The periprosthetic capsule requires meticulous gross examination. If the specimen is a total capsulectomy with the implant in place, the recommended procedure is to first ink the outer margins by designated quadrants—particularly if oriented by the surgeon—and open the capsule. Any fluid should be collected and submitted for cytology evaluation as mentioned above. The implant is then removed and examined for integrity and gross appearance. Then the fibrous capsule is opened flat and carefully inspected for the presence of any attached fibrinous or necrotic material or a mass. The capsule is pinned to a flat surface (luminal side up) and fixed overnight in neutral buffered formalin (Figure 16A). Findings such as fibrinous or necrotic material on the inner capsule surface, irregularities, and/or wall thickenings must be sampled. Because of the multifocality of BIA-ALCL, adequate and thorough sampling is required. For the optimal handling of these specimens, it is recommended that at least two random sections of each of the six aspects of the capsule (anterior, posterior, medial, lateral, superior, inferior) are included to have at least 12 tissue sections for evaluation. All sections should be at least 2 cm long and should be submitted “on edge” to make it possible to assess the largest surface area of the luminal side of the capsule [169]. This technique increases the chance of locating minute or microscopic lesions that may have been missed or cannot be seen grossly. If a rim of breast tissue or mural lesions/nodules are present, they should be sampled extensively. An assessment of the stained margins is also recommended [30,168,169]. Approximately 20% of patients with BIA-ALCL present initially with associated ipsilateral axillary lymphadenopathy. A lymph node biopsy should be obtained in such cases and handled just as any other lymphoma protocol [170].

### 4.6. Pathology

BIA-ALCL has unique cytoarchitectural features that differ from systemic ALK- ALCL and pc-ALCL. A noteworthy difference is that in BIA-ALCL, the first specimen to be evaluated is usually the peri-implant fluid material rather than tissue sections [169]. The neoplastic cells in the fluid may be scant or numerous and intermingled with fibrinoid and necrotic material and with small lymphocytes, macrophages, and plasma cells [171]. The neoplastic cells are large (4–5X larger than a small lymphocyte) with significant atypia and may have cytoplasmic vacuoles. The nucleus is usually bilobed and hyperchromatic, with prominent nucleoli. In some cases, however, the nucleus is markedly displaced to the periphery to resemble a signet-ring cell. “Hallmark” cells are seen in up to two-thirds of cases (Figure 16B,C).

**Figure 17:**
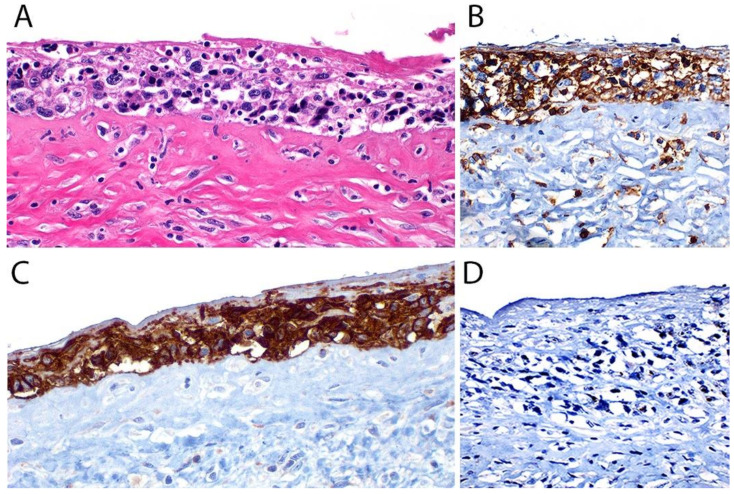
(**A**) Histologic section of the breast fibrous capsule shows numerous atypical large lymphoid cells with pleomorphic nuclei admixed with fibrino-necrotic debris on the luminal side. These cells are positive for (**B**) CD45 and (**C**) CD30, and negative for (**D**) ALK. CD30 is also positive in the fibrino-necrotic debris.

Histologic sections of the capsule show variable amounts of lymphoma cells lining the luminal side with identical morphology to that previously described in ALCL (Figure 17A). The lymphoma cells may or may not be embedded in variable amounts of coagulative necrosis with numerous “ghost” tumor cells, and some cases may only contain necrotic tumor cells. For this reason, it is important to keep a high threshold of suspicion for BIA-ALCL in capsulectomy specimens that have been resected—particularly if there is a history of an effusion—and they feature an “eosinophilic acellular lining” or “fibrin” on the luminal surface. The lymphoma cells may be confined to the luminal side, may infiltrate the capsule wall, or may extend beyond the capsule and invade the surrounding breast parenchyma. An evaluation of the extent of capsular invasion is required for proper staging (see below).

#### 4.6.1. Immunohistochemistry

The immunophenotype of the lymphoma cells is identical to ALK- ALCL and pc ALCL. BIA-ALCL cells are strongly positive for CD30, variably positive for CD45, and are negative for ALK (Figure 16D and Figure 17B–D). The neoplastic cells are variably positive for EMA, CD4, CD43, and TIA-1 as well as for the T-cell markers CD2, CD3, and CD45RO, while negative for CD20, PAX5, and CD68 [30]. Strong positivity for CD30 is very useful to confirm the diagnosis both in a cell block and while observing the luminal side of a capsule, particularly at the early stages (T1-T2, see below). A negative CD30 excludes the diagnosis of BIA-ALCL. In a similar fashion to its systemic and primary cutaneous counterparts, BIA-ALCL is not associated with EBV infection. Axillary lymph node involvement by BIA-ALCL can exhibit a sinusoidal, interfollicular, and/or perifollicular pattern. Nodal involvement is frequently focal or partial and the median tumor burden is ~30%. A small percentage of cases demonstrate a diffuse pattern with a complete effacement of the nodal architecture [156].

#### 4.6.2. Staging

**Figure 18:**
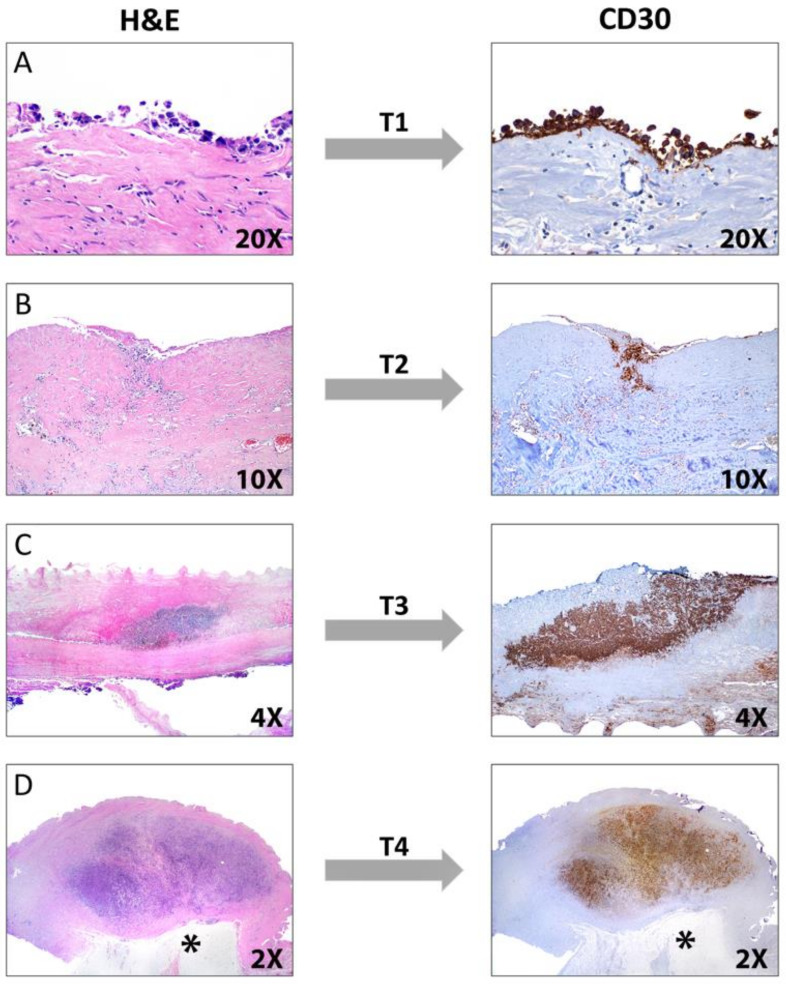
Pathologic tumor stage (T) used in breast implant-associated ALCL. Left panel: hematoxylin and eosin stain, H&E. Right panel: CD30 immunostain. (**A**) T1, the lymphoma cells are present only in the effusion and/or confined to the lumen of the fibrous capsule. (**B**) T2, sparse neoplastic cells infiltrate the capsule. (**C**) T3, solid aggregates or sheets of lymphoma cells within the capsule; (**D**) T4, a solid nodule of lymphoma cells occupies the entire capsule thickness and approaches the adipose tissue and breast parenchyma (asterisk). Immunohistochemistry for CD30 is more accurate to evaluate the level of infiltration of the capsule.

Unlike most NHLs, BIA-ALCL should be staged following the TNM guidelines of the American Joint Committee on Cancer (AJCC). In this lymphoma, T corresponds to the depth of the tumor invasion of the peri-implant fibrous capsule, N to the infiltration of the regional lymph nodes, and M to distant metastases [170,172]. The pathologist plays a key role in the pathologic staging of BIA-ALCL. Stage T1 corresponds to the earliest pattern, characterized by the presence of only a positive effusion, and/or superficial and/or focal involvement of the inner capsule surface (Figure 18A). Necrotic and fibrinous material, karyorrhectic debris, and “ghost” neoplastic cells are observed. Viable lymphoma cells are sparse and arranged in a single layer or are isolated and “floating” very close to the surface. These cells are strongly positive for CD30 as is the necrotic material. Stage T2 consists of the infiltration of the fibrous capsule by isolated lymphoma cells intermingled with other inflammatory cells such as macrophages, small lymphocytes, and plasma cells (Figure 18B). In stage T3, solid aggregates or sheets of lymphoma cells are observed within the capsule (Figure 18C). Finally, in stage T4, ≥1 neoplastic nodule(s) are observed throughout the capsule thickness and beyond, sometimes involving adjacent tissues or surgical margins (Figure 18D). In this pattern, there may be nodules of lymphoma cells surrounded by fibrous bands and admixed with small lymphocytes, plasma cells, and eosinophils, a background identical to that of nodular sclerosis CHL [172]. CD30 immunostaining is required to adequately evaluate the extent of capsular invasion by lymphoma. Importantly, the pathology report must include the status of the resection margins [168,170].

### 4.7. Differential Diagnosis

An implant-associated reactive inflammatory change is the main alternative diagnosis of BIA-ALCL since atypical-appearing macrophages, multinucleated giant cells, myofibroblasts, and immunoblasts are morphologic mimics of ALCL cells and are also present in an effusion and on the surface and within the fibrous capsule [173]. Pathologists should be aware that immunoblasts (B and T), plasma cells, and stromal cells can express CD30, but usually not at the level of intensity as seen in BIA-ALCL [174]. The diagnosis of BIA-ALCL requires the presence of characteristic anaplastic lymphoma cells strongly and diffusely expressing CD30 in a membranous and paranuclear dot pattern, in addition to the expression of T-cell markers, CD4, and TIA-1. CD30 is extremely helpful to evaluate cases with extensive tumor necrosis since the necrotic material is strongly and diffusely positive for this marker.

As mentioned above, BIA-ALCL extending beyond the fibrous capsule can mimic nodular sclerosis CHL. The diagnosis of CHL can be excluded based on the lack of PAX5 in the lymphoma cells. Other markers that may be useful to separate these entities include CD15, CD43, CD45, and EBER. CHL is usually positive for CD15 and negative for CD43 and CD45 with the opposite labelling pattern in BIA-ALCL. The localization to the breast around an implant capsule as well as the prior presence of an effusion with lymphoma cells should be confirmatory of BIA-ALCL and not CHL. Moreover, CHL is exceedingly rare at this extranodal location [156,175].

The extension of systemic ALK- ALCL to the breast should always be ruled out before considering the possibility of BIA-ALCL. In this instance, there is usually a known history of systemic disease. If a patient has breast implants and has systemic ALK- ALCL and the capsule is involved, this clinical presentation should be considered BIA-ALCL with progression to systemic disease. Likewise, nodal involvement by BIA-ALCL cannot be distinguished from systemic ALK- ALCL involving an axillary lymph node. Clinical correlation is the only way to determine the origin of the nodal disease.

Tumors that can occur in patients with breast implants other than BIA-ALCL include other NHLs, breast carcinoma, and metastatic carcinoma to peri-implant breast tissue [145,176,177,178]. Recently, there have been a few reported cases of EBV-associated DLBCL arising within the fibrous capsule of breast implants, expanding the spectrum of BIA-lymphoproliferative disorders [176,177,178]. The recurrence of a previous breast carcinoma should always be considered in the differential diagnosis of BIA-ALCL with breast parenchymal involvement, particularly in cases of reconstructive breast implant surgery. An adequate morphologic evaluation complemented with a proper set of immunohistochemical stains (B-cell markers, T-cell markers, keratins, CD30, ER, PR, ALK, EBER) are usually sufficient to establish a correct diagnosis.

### 4.8. Genetic Findings and Molecular Alterations

Only a few reports of cytogenetic findings in BIA-ALCL are available to date. Cytogenetic studies performed in BIA-ALCL cell lines (TLBR-1, TLBR-2, and TLBR-3) have shown a complex karyotype and the absence of chromosomal abnormalities associated with other lymphomas, including systemic ALK- ALCL and pc-ALCL [179]. In addition to complex karyotypes, losses of chromosomes 1p, 10p, 20, and the gain of 19p have been observed in few cases [30,180]. The gain of 19p, the region where a JAK kinase is encoded, could help to clarify the pathogenesis of this entity, as this protein is involved in the phosphorylation of STAT1 and STAT3 [30,181].

Unlike systemic ALK- ALCL and pc-ALCL, no rearrangements of ALCL-associated genes such as *ALK*, *DUSP22*, and *TP63* have been detected in BIA-ALCL and therefore, this subtype can be considered a triple-negative ALCL [40,182]. Potential molecular drivers of BIA-ALCL include the JAK/STAT signaling pathway and *MYC* and *TP53* deregulation. JAK/STAT point mutations are the most frequent in this lymphoma [183]. Alterations in members of the JAK/STAT signaling pathway have also been described in systemic ALCL (ALK+ and ALK-) and pc-ALCL, as mentioned in other sections of this review [181]. The activation of this pathway via STAT3 phosphorylation is almost always present in BIA-ALCL. *STAT3* mutations are found in 26% of cases [184,185]. The *STAT3* S614R is the predominant point mutation (67%), and it affects the SH2 domain leading to constitutive STAT3 activation and protein phosphorylation [182]. *JAK1* mutations occur in 13% of cases. The *JAK1* G1097V mutation is the only one that has been reported in BIA-ALCL to date. This mutation results in increased protein function and a subsequent increase in STAT3 phosphorylation. The *JAK3* germline mutation V722I is considered a genetic predisposing factor for BIA-ALCL since it creates a positive feedback loop in JAK-STAT mutations [181,186]. All these mutations promote cell proliferation and survival as well as oncogene activation resulting in clonal expansion and tumor development, and they may benefit the growth of BIA-ALCL cells in the inflammatory environment surrounding the implant. This process has been described in cancers associated with persistent chronic inflammation [187].

Another gene implicated in the pathogenesis of BIA-ALCL is *TP53*. When this gene is mutated, there is an abnormal response to DNA damage. Moreover, some *TP53* germline mutations appear to predispose individuals to BIA-ALCL, as in the case of Li-Fraumeni syndrome [188]. Although *TP53* mutations and/or deletions have been associated with genomic instability, which could explain its presence in patients with BIA-ALCL, the specific risk for developing BIA-ALCL in the presence of *TP53* genetic alterations has not been defined [188].

*MYC* gene alterations (mainly copy number gains) have also been reported in BIA-ALCL [181]. Although *MYC* overexpression is strongly associated with the pathogenesis of systemic ALK- ALCL due to overstimulation of T-cells by *IRF4*, its effect on the pathogenesis of BIA-ALCL has not been fully elucidated [189].

Lastly, a possible contributor to genetic predisposition for BIA-ALCL has recently been suggested. It appears that mutations in the *BRCA1* and *BRCA2* genes increase the risk of developing BIA-ALCL from 1 in 7507 to 1 in 1551, but this finding is not definitive and more studies are needed to validate these observations [190].

## 5. Conclusions

ALK- ALCL is a rare subtype of TCL with overall well-defined histopathologic features. However, the differential diagnosis with CD30+ large PTCL, NOS and CHL with expression of T-cell markers may occasionally overlap, although currently this distinction may not be clinically relevant at least for those cases of PTCL, NOS. More importantly, unusual histologic variants or cases with a “null” phenotype can be missed or confused with several other hematopoietic and non-hematopoietic malignancies. For this reason, we cannot stress enough the need to think about the possibility of ALK- ALCL and perform immunohistochemistry for CD30 and CD43 in any poorly differentiated neoplasm that has failed to demonstrate any specific lineage marker to not miss potential unusual cases of this TCL. This will have a significant impact on the treatment and prognosis of these patients. Moreover, clinicopathologic correlation is mandatory to distinguish pc-ALCL and BIA-ALCL from systemic ALK- ALCL. The recognition of novel genetic alterations in a subset of ALK- ALCLs, such as *DUSP22* and *TP63* rearrangement, *MSC^E116K^*, and mutations in molecules of the JAK/STAT pathway now permit us to stratify these cases into those with a better or worse prognosis, which is a giant leap in the field of TCLs and hematology/oncology. It is very likely that in the near future the detection of these—and not yet discovered—mutations will become crucial in the identification of potential therapeutic targets.

## Authors Contributions

Conceptualization: S.P.-O. All authors (S.P.-O., C.O.-H., A.A.C.-Z. and A.Z.-O.) have drafted, revised, and approved the submitted version of this manuscript. All authors have read and agreed to the published version of the manuscript.

## Figures and Tables

**Table 1:** Differential diagnosis of ALK- ALCL and other hematopoietic tumors.

Entity	Morphologic Features	CD30	CD15	CD4	CD8	Other T-Cell Markers: CD2, CD3, CD5, CD7	Cytotoxic Markers:Granzyme B, TIA-1, Perforin	MUM1	ALK	PAX5	EBER ISH	EMA, Other
ALK- ALCL	Hallmark cells Pleomorphism Sinusoidal pattern May show starry-sky pattern *DUSP22-*rearranged: >Doughnut cells <Pleomorphism	+ (>75% of cells)	Usually -rarely +	Usually +	Usually -	Variable for all	Usually + except in *DUSP22*-rearranged cases	+	-	Usually -Very rarely + (*PAX5* extracopies)	-	Variable + EMA (40%)
ALK+ ALCL	Hallmark cells Pleomorphism Doughnut cells Sinusoidal pattern	+ (>75% of cells)	- Very rarely +	Usually +	Usually -	Variable for all	Usually +	+	+ variable patterns	Usually -Very rarely + (*PAX5* extracopies)	-	Usually + EMA (>80%)
Large cell PTCL, NOS	Usually no hallmark cells Variable pleomorphism	Variable <75% of cells	Usually -rarely +	Variable	Variable	Variable	Usually - unless CD8+	Variable	-	-	- Rarely +	- EMA If CD30+ difficult to distinguish from ALK- ALCL
CHL	Sclerosis and cellular nodules Polymorphic infiltrate Usually no hallmark cells Usually no sinusoidal patternSolid growth in syncytial variant	+	Usually + (60–70%)	Usually – Few +	Usually – Few +	Usually – Few variable	Variable	+	-	Dim +	Variable NS-CHL (20%) MC-CHL (80%)	- EMA
Anaplastic LBCL	Similar to ALCL, usually no hallmark cells May show sinusoidal pattern	+ (30% of cases)	-	-	-	-	-	Variable	- unless ALK+ LBCL (cytoplasmic granular)	+ Other B-cell markers +	- unless EBV+ LBCL	- EMA unless ALK+ LBCL B-cell markers variable or–in ALK+ LBCL
Myeloid/monocytic sarcoma	Usually no hallmark cells Variable pleomorphism May show starry-sky pattern	Usually -	+	+ if monocytic	-	-	Variable	-	-	- + if t(8;21)	-	- EMA + CD13, CD14, CD64, MPO, lysozyme
Histiocytic sarcoma	Usually no hallmark cells Variable pleomorphism Usually no sinusoidal pattern	-	Variable	+	-	-	Variable	-	-	-	-	- EMA + monocyte/ macrophagemarkers CD68, CD163

Abbreviations: ALK: anaplastic lymphoma kinase; ALCL: anaplastic large cell lymphoma; EBER ISH: Epstein-Barr virus encoded RNA in situ hybridization; EMA: epithelial membrane antigen; PTCL, NOS: peripheral T-cell lymphoma, not otherwise specified; CHL: classic Hodgkin lymphoma; NS-CHL: nodular sclerosis CHL; MC-CHL: mixed cellularity CHL; LBCL; large B-cell lymphoma.

**Table 2:** Differential diagnosis of ALK- ALCL and non-hematopoietic tumors (lymph node metastasis or extranodal mass).

Entity	Morphologic Features	CD43/ CD45	CD30	T-Cell Markers	ALK	CKs	p63	EMA	Melanoma Markers: S100, MART1, HBM-45, SOX10	OCT3/4, SALL4	INI1/SMARCB1	Desmin	Vascular Markers, Other
ALK- ALCL	Hallmark cells PleomorphismSinusoidal pattern May show starry-sky pattern *DUSP22-*rearranged: >Doughnut cells <Pleomorphism	Usually + Few -	+	Variable, usually CD4+ All may be –(null phenotype)	-	- Very rarely +	Variable, + in *TP63*-rearranged cases	Variable + EMA (40%)	-	-	Retained	-	-
Carcinoma	May show hallmark-like cells Variable pleomorphism Sinusoidal pattern	-	-	-	-	+	+ if squamous cell carcinoma	+	-	-	Retained	-	- Other markers may be + depending on subtype: TTF1, PAX8
Embryonal carcinoma	May show hallmark-like cells Variable pleomorphism Pseudoglandular, alveolar, solid patterns	-	+	-	-	+	-	+	-	+	Retained	-	-
Melanoma	May show hallmark-like cells Variable pleomorphism Sinusoidal pattern	-	- Very rarely +	-	-	-	-	-	+	-	Retained	-	-
Epithelioid sarcoma, other rhabdoid tumors	May show hallmark-like cells Variable pleomorphism Nodular and solid growth, central necrosis Subcutaneous, soft tissue mass	-	-	-	-	+	-	+	-	-	Loss	-	+ ERG
Alveolar soft part sarcoma	May show hallmark-like cells Variable pleomorphism Nodular and alveolar growthSoft tissue mass	-	-	-	-	-	-	-	-	-	Retained	-	- + TFE3 and cathepsin K
Epithelioid myofibroblastic sarcoma	Variable pleomorphism Intraabdominal mass Very rare	-	+	-	+ nuclear membrane or perinucleolar	-	-	-	-	-	N/A	+	- - caldesmon variable + SMA

Abbreviations: ALK: anaplastic lymphoma kinase; ALCL: anaplastic large cell lymphoma; CKs: cytokeratins; EMA: epithelial membrane antigen; SMA: smooth muscle actin.

**Table 3:** Differential diagnoses of primary cutaneous ALCL.

Entity	Main Clinical and Histopathologic Features
Systemic ALK+ ALCL with cutaneous involvement	Cutaneous involvement occurs in up to 60% of cases Peripheral lymph nodes and extranodal sites also affected CD30+, ALK+, EMA+ (strong) *ALK* gene rearrangement
Systemic ALK- ALCL with cutaneous involvement	Peripheral lymph nodes and/or extranodal sites affected Morphologically and immunophenotypically indistinguishable from pc-ALCL
MF with large cell transformation	Clinical history of MF with patches, plaques, and tumors Histological characteristics of MF may be present pan T-cell antigens +, may be CD7- and/or CD5- CD30 may be variable +, GATA3+
Classic Hodgkin lymphoma with cutaneous involvement	Peripheral lymphadenopathy, very rarely presents with skin involvement PAX5+ (weak), often CD15+, lack of CD45 Often Epstein-Barr virus + (EBER ISH, LMP-1) T-cell markers usually -
Lymphomatoid papulosis (LyP) type C	Multiple papular and papulonecrotic eruptions < 10 mm History of spontaneous regression favors LyP Morphologically and immunophenotypically indistinguishable from pc-ALCL
PTCL, NOS, with cutaneous involvement	B symptoms and evidence of disseminated lymphadenopathy Very rarely presents with skin involvement CD30- or weakly +
Subcutaneous panniculitis-like TCL	Median age: 35 years, F > M Infiltrate confined to subcutaneous tissue with fat necrosis; dermis and epidermis typically uninvolved CD30-, CD8+, cytotoxic proteins +
Primary cutaneous gamma-delta TCL	Common hemophagocytic syndrome Three histologic patterns: epidermotropic, dermal, and subcutaneous Apoptosis, necrosis, and angioinvasion present TCRγ/δ+, CD2+, CD5-, CD7+/-, CD56+, cytotoxic proteins +, CD30 variable
Primary cutaneous CD8+ aggressive epidermotropic cytotoxic TCL	Extensive ulcerated cutaneous nodules with an aggressive clinical course Epidermotropic proliferation with pagetoid pattern Ulceration, necrosis, and angioinvasion CD3+, CD8+, cytotoxic proteins +, TCRαβ+, CD2-, CD30-, high Ki-67
Primary cutaneous CD4+ small/medium T-cell lymphoproliferative disorder	Solitary plaque or nodule in face, neck, or upper trunk <30% of large cells CD3+, CD4+, cytotoxic proteins -, CD30- TFH phenotype: PDL-1 (CD279)+, bcl6+, CXCL13+

Abbreviations: ALK: anaplastic lymphoma kinase; ALCL: anaplastic large cell lymphoma; TCL: T-cell lymphoma; PTCL, NOS: peripheral TCL, not otherwise specified; MF: mycoses fungoides; EBER ISH: Epstein-Barr virus encoded RNA in situ hybridization; LMP-1: latent membrane protein-1 of EBV; THF: T follicular helper; pc: primary cutaneous; F: females; M: males.

**Table 4:** Genetic abnormalities in pc-ALCL.

Genetic Alteration	Frequency
Clonal T-cell receptor gamma genes	~90%
*ALK* and t(2;5) translocation	Absent in 80–90%
Gains of 7q31, 17q, and 21 Losses of 3p, 6q16–6q21, 6q27, 8p, and 13q34	~50%
Rearrangement of the *DUSP22-IRF4* locus on 6p25.3	~25% Detection of 6p25.3 rearrangement by fluorescence in situ hybridization is highly specific for pc-ALCL (specificity of 99%)
